# Identification of food and nutrient components as predictors of *Lactobacillus* colonization

**DOI:** 10.3389/fnut.2023.1118679

**Published:** 2023-04-21

**Authors:** Sharon C. Thompson, Amanda L. Ford, Elijah J. Moothedan, Lauren S. Stafford, Timothy J. Garrett, Wendy J. Dahl, Ana Conesa, Claudio F. Gonzalez, Graciela L. Lorca

**Affiliations:** ^1^Department of Microbiology and Cell Science, Genetics Institute, Institute of Food and Agricultural Sciences, University of Florida, Gainesville, FL, United States; ^2^Department of Food Science and Human Nutrition, Institute of Food and Agricultural Sciences, University of Florida, Gainesville, FL, United States; ^3^Department of Pathology, Immunology, and Laboratory Medicine, College of Medicine, University of Florida, Gainesville, FL, United States; ^4^Institute for Integrative Systems Biology, Spanish National Research CouncilValencia, Spain

**Keywords:** lactic acid bacteria, dietary intake, microbiome, probiotic, *Lactobacillus johnsonii* N6.2, machine learning, multivariate analysis

## Abstract

A previous double-blind, randomized clinical trial of 42 healthy individuals conducted with *Lactobacillus johnsonii* N6.2 found that the probiotic’s mechanistic tryptophan pathway was significantly modified when the data was stratified based on the individuals’ lactic acid bacteria (LAB) stool content. These results suggest that confounding factors such as dietary intake which impact stool LAB content may affect the response to the probiotic treatment. Using dietary intake, serum metabolite, and stool LAB colony forming unit (CFU) data from a previous clinical trial, the relationships between diet, metabolic response, and fecal LAB were assessed. The diets of subject groups with high vs. low CFUs of LAB/g of wet stool differed in their intakes of monounsaturated fatty acids, vegetables, proteins, and dairy. Individuals with high LAB consumed greater amounts of cheese, fermented meats, soy, nuts and seeds, alcoholic beverages, and oils whereas individuals with low LAB consumed higher amounts of tomatoes, starchy vegetables, and poultry. Several dietary variables correlated with LAB counts; positive correlations were determined for nuts and seeds, fish high in N-3 fatty acids, soy, and processed meats, and negative correlations to consumption of vegetables including tomatoes. Using machine learning, predictors of LAB count included cheese, nuts and seeds, fish high in N-3 fatty acids, and erucic acid. Erucic acid alone accurately predicted LAB categorization, and was shown to be utilized as a sole fatty acid source by several *Lactobacillus* species regardless of their mode of fermentation. Several metabolites were significantly upregulated in each group based on LAB titers, notably polypropylene glycol, caproic acid, pyrazine, and chondroitin sulfate; however, none were correlated with the dietary intake variables. These findings suggest that dietary variables may drive the presence of LAB in the human gastrointestinal tract and potentially impact response to probiotic interventions.

## Introduction

Probiotics and fermented foods have been studied and used throughout human history for their various beneficial properties. Such microorganisms have been shown to have several benefits including homeostasis of the gut microbiota and production of beneficial molecules such as short chain fatty acids and antimicrobials, albeit mostly in animal models (1–3). Animal studies are routinely used to gather pre-clinical data due to the ability to perform interventions under controlled conditions and ease of genetic manipulation for mechanistic studies. In recent years, the clinical study of several potentially probiotic strains has intensified on a spectrum of human diseases. However, despite often promising results in animal models, the beneficial effects are often not translated into clinical studies. Human interventions present several challenges including confounding factors despite efforts to randomize and control them. Confounding factors may include duration of the intervention, methods used for microorganism detection, genetics, and diet. For example, in a study of the efficacy of *Bifidobacterium animalis* spp. *Lactis* B94 in adults with Prader-Willi Syndrome, it was expected that the administration of the bacterium would result in alterations in stool frequency, gastrointestinal symptom rating scale (GSRS) syndrome, and microbiota composition. However, a delayed response in certain stool forms was observed, leading to the hypothesis that length of intervention and washout periods may have affected detection of delayed probiotic responses (4). Similarly, *Lactobacillus gasseri* BNR17 was shown to have a beneficial effect in models of obesity in murine models (5); however, no changes in body weight were found in a human clinical intervention when compared to the control cohort (6, 7).

In contrast, in our studies with *Lactobacillus johnsonii* N6.2, we were able to determine that mechanistic pathways found in animal studies partially translated into human subjects in an intervention study in healthy adults. In animal studies, the administration of *L. johnsonii* N6.2 resulted in reduced mRNA expression and the enzymatic activity of Indoleamine 2,3 dioxygenase (IDO) resulting in modifications in the tryptophan to kynurenine ratios. These alterations were correlated with a Th17 bias (8, 9). In the clinical trials it was found that while the administration of *L. johnsonii* N6.2 resulted in a reduction in the percentage of CD4+ lymphocytes and GSRS scores, fluctuations in the levels of tryptophan and kynurenine/tryptophan ratios were only observed in subjects with increasing stool counts of lactic acid bacteria (LAB) over time (10). Given these results, we hypothesized that dietary intake is a variable that may contribute to the ability of probiotic strains to colonize or exert its beneficial effects.

Diet has been indicated as an important driver for fluctuations in the microbiota. For example, in a clinical intervention of diets with varying total amounts of fats, subjects consuming high fats led to increased abundance of *Bifidobacterium*, while low total fat consumption led to increased *Faecalibacterium prausnitzii* (11). In a murine study, intervention with unsaturated fats from fish oils were shown to result in increased relative abundance of *Acinetobacteria*, lactic acid bacteria, and *Verrucomicrobia* (12). Further, research has expanded to the evaluation of the role of dietary interventions on the interaction between microbiota and the human immune status. It was recently reported that a high-fermented-food diet resulted in an increase in microbiota diversity and a decrease in inflammatory markers (13). Early studies on the infant microbiota determined that the composition of the microbiota as well as its impact in health outcomes may be influenced by certain components present in the food intake, termed prebiotics (14). Some of the most well-known and understood prebiotics are human milk oligosaccharides (HMO), which are known to modulate the presence of *Bifidobacterium* in the human gut (15). While less understood, prebiotic phytophenols abundant in fruits and vegetables also have profound changes on the gut microbiota. We have previously reported that the combined administration of *L. johnsonii* N6.2 and blueberry extracts, high in dietary phytophenols, resulted in synergistic effects and potentiated their beneficial effect in rodents under a high fat diet (16, 17). While there is a breadth of information regarding the impact of various dietary interventions on the intestinal microbiome, there is a lack of study on the impact of diet during clinical intervention of probiotics. Based on these observations, in this clinical study, dietary intake surveys of healthy patients were recorded without alteration or intervention to the diet. We then utilized multidimensional statistical analyses to model and determine the relationships between habitual dietary intake of healthy subjects and the presence and efficacy of probiotic intervention of *L. johnsonii* N6.2.

## Materials and methods

### Clinical trial

Secondary analysis was performed on data obtained from a clinical trial conducted on 42 healthy adults which evaluated the safety and immune response to daily consumption of *L. johnsonii* N6.2 (13, 14). The clinical trial consisted of three periods, a 1-week baseline, 8-week intervention, and 4-week washout. During the intervention period, subjects received either 1 capsule per day of 10^8^ colony-forming units (CFU) of *L. johnsonii* N6.2 or placebo (skim milk) for 8 weeks. The study was approved by the Institutional Review Board 1 (# 201400370) at the University of Florida. Total LAB counts were analyzed on stool samples collected at baseline, week 2, 4, and 8 of treatment, and a final collection after washout (week 12). Details of the stool collection methods can be found in Marcial et al. (10). LAB were enumerated by plating fresh stool samples (1 g) diluted in phosphate buffer solution (pH 7.4) on acidified MRS agar media (pH 5.5 ± 0.1) and incubating 48 h at 37°C under microaerobic conditions. Enumeration values were referred to as CFU per wet gram stool (CFU/g).

### Dietary intake surveys

Study participants recorded their dietary intake for 2 days during each period using the online software Automated Self-Administered 24-Hour (ASA24®) Dietary Assessment Tool (version 2018) for a total of up to six recalls (13). Participants had 24 h to complete their dietary recall from the date of the intake and were not alerted beforehand of which dates would be scheduled. Participants who completed less than 3 days of intake were excluded from statistical analysis (n = 5). See Table 1 for the adjusted demographics of the data analyzed.

**Table 1:** Study adjusted demographics of individuals included in analyses.

Measure	Intervention group		LAB CFU/g stool	
	Placebo (*n* = 18)	Ljo^a^ (*n* = 19)	High (*n* = 18)	Low (*n* = 10)
Gender (M/F), *n*	6/12	4/15	4/4	4/6
Age, years median (range)	21 (18–48)	23 (18–36)	23 (18–48)	21 (18–26)
Race/ethnicity, *n* (%)				
Asian	11%	16%	22%	10%
African American	6%	5%	11%	0%
Hispanic	17%	21%	17%	10%
White	83%	58%	50%	90%
Other^b^	0%	16%	5%	0%
BMI, mean (SD)	23.9 ± 5	23.6 ± 4.5	23.2 ± 4.5	24.1 ± 3.9
Blood Pressure (mean mm Hg)	117/75	118/74	116/74	119/74
Compliance (%)	90 ± 0.1	90 ± 0.1	90 ± 0.1	90 ± 0.1

a *Lactobacillus johnsonii* N6.2.

b Participants who classified themselves as other included *n* = 2 Hawaiian and *n* = 1 unknown.

### Data assessment and stratification

For initial analyses, the data were separated by intervention, participants receiving *L. johnsonii* N6.2 (*n* = 20), and the placebo group (Pla; *n* = 18; See Supplementary Figure S1). The data were then stratified based upon the participants’ stool counts of LAB. Three groups were previously defined in Marcial et al. (10) as subjects with high counts of LAB throughout the study (>10^5^ CFU/g) termed High LAB (H-LAB, *n* = 18), subjects that increased in counts of LAB over the course of the study (10^4^ to 10^8^ CFU/g) termed Low-to-High LAB (LH-LAB, *n* = 10), and subjects with low counts of LAB throughout the study (<10^5^ CFU/g) termed Low LAB (L-LAB, *n* = 10). Associations between dietary intake, CFU/g wet stool LAB, and serum untargeted metabolomic data of these subjects were then explored based on this stratification. Each stratification and its purpose are described for each separate set of data and analysis in the methods below.

### Diet analysis

Individuals were grouped based on their intervention group (*L. johnsonii* N6.2 versus placebo) and by LAB counts in separate analyses (Supplementary Figure S1). Individual average nutrient intakes were adjusted to estimate the habitual diet, defined as the average food consumption over a period of time over which a dietary pattern is maintained (18). To remove within-person variance, the equation put forward by the US National Academy of Science Subcommittee on Criteria for Dietary Evaluation was used,


$$\begin{array}{l} \text{Adjusted value for the nutrient}=\text{average}\\ \;+(X_{i}-\mathrm{average})*S_{b}/S_{\text{obs}} \end{array}$$

where the average is the mean value for the group, *x*_i_ is the value observed for each individual, *S_b_*/*S_obs_* is the inverse of the within-person variance (19). Finally, the residual method was used to normalize for energy intake and gender (20). Using the adjusted average intake values and the stratifications described, several multivariate analyses were performed to evaluate and describe dietary patterns and differences among the groups of individuals. The nutrition data were also stratified by groups as defined by ASA24: macronutrients, vitamins, minerals, other, grains, meats (referred to as proteins in this text), vegetables, fruits, dairy, and “extra” which includes discretionary oils and fats (21). Total fats, within macronutrients, are subdivided into saturated fatty acids, monounsaturated fatty acids, and polyunsaturated fatty acids (Supplementary Table S1).

### Statistical analyses

One-way analysis of variance (ANOVA) was used to compare the adjusted average intake of all nutrients and food groups for individuals in the H-and L-LAB groups. Linear regressions were completed with the adjusted intake value of each nutrient and food group and the log (CFU/g wet stool) lactic acid bacteria. After multiple test correction, significance was considered as a *value of p* of less than 0.05. Principal component analysis (PCA) plots were assembled using the R package FactoMineR (22). Visualization of eigenvalues and PCA plots were done using corrplot package (21). Linear regression was used in the R stats package to complete principal component regression with log (CFU/g wet stool) LAB used as the dependent variable and PC’s from previous PCA used as independent variables (23). Beta coefficients and R^2^ values were calculated and recorded for each regression. Significance for all analyses was considered as a *value of p* of less than 0.05.

### Machine learning

Extreme gradient boosting was utilized for the categorical prediction of LAB grouping using the R package xgboost (24). Each model was trained separately with groups of variables (dietary intake as listed in Supplementary Table S1). The depth of the trees was set to three for each model for maximum accuracy. The minimum testing root-mean-square deviation (RSME) was determined for each model and used to set the optimal number of rounds of boosting for reduction of overfitting. Percent accuracy of model predictions, value of ps, and variable importance were determined for each model. Next, classification type random forests were generated using the R package randomForest to predict individual classification of H-and L-LAB groups based on nutrition data (22). Each model was trained separately with groups of variables (dietary intake as listed in Supplementary Table S1). Groups included macronutrients, vitamins, minerals, saturated fats, polyunsaturated fats, monounsaturated fats, grains, vegetables, fruits, proteins, dairy, and food additives. Each model was improved by optimizing the number of variables randomly sampled as candidates at each split, referring to each random subset of variables, within the random forest tree, known as the mtry value, and removing variables to reduce error rate. Each model was repeated 500 times with distribution of predictive rates, variable importance, and frequency at which a variable is predictive within the models calculated for each model. Variable importance is presented by mean decrease Gini value which is a measure of how each variable contributes to the homogeneity of the random forest. A higher the value of mean decrease Gini score equates to a higher level of importance within the model, thus allowing the ranking of usefulness of variables. For both modeling techniques, the data were separated into two groups, 70% used to train the data set (*n* = 20), and 30% used to test the dataset (*n* = 8). A total of 17 models were then refined, one per dietary group, and run 500 times to determine their accuracy and reproducibility of predictions. Potential biomarkers were chosen based upon their decrease gini value, highest frequency of prediction, and importance within a model of >85% accuracy of prediction.

### Metabolomics

Global metabolomics data was obtained as described in Marcial et al. (10) For identification of the features, the mass-to-charge ratios and ionization mode were used to search on-line databases such as the Human Metabolome Database (25).

### Microbial growth kinetics experiments

The bacterial strains used are found in Supplementary Table S4. All strains were first activated in MRS-T (10 g/L peptone, 10 g/L beef extract (Bacto), 5 g/L yeast extract (Bacto), 20 g/L glucose, 2 g/L di-potassium hydrogen phosphate, 5 g/L sodium acetate, 2 g/L ammonium citrate tribasic, 0.2 g/L magnesium sulfate, 0.05 g/L manganese sulfate, 1 ml/L Tween 80). To evaluate the growth requirement of fatty acids, growth experiments were performed in four variations of MRS-T. First, MRS-NT was formulated by removing Tween 80 from MRS-T. Then, oleic or erucic acid was added at 0.1% (v/v) to make MRS-O or MRS-E, respectively. MRS-T with 0.1% DMSO was used as a vehicle control (MRS-TD). For growth curves, cells were subcultured twice in MRS-T for 16 h at 37°C. From the second activation culture, cells were pelleted by centrifugation at 3,000× *rpm* for 8 min and washed with MRS-NT. Cells were then suspended in MRS-NT and used to inoculate the media with the different fatty acids at an initial optical density (OD_600_) of 0.05 in biological triplicates. Cells were incubated at 37°C and OD_600_ recorded at 2 h intervals for a total of 18 h. Growth kinetics μmax and duplication time (DT) were estimated using a linear model of exponential phase. Statistics were performed in R as described previously by ANOVA and t-test, with *p* < 0.05 being considered statistically significant.

## Results

### Principal component analysis identified dietary components enriched in subjects with high or low LAB counts

**Figure 1:**
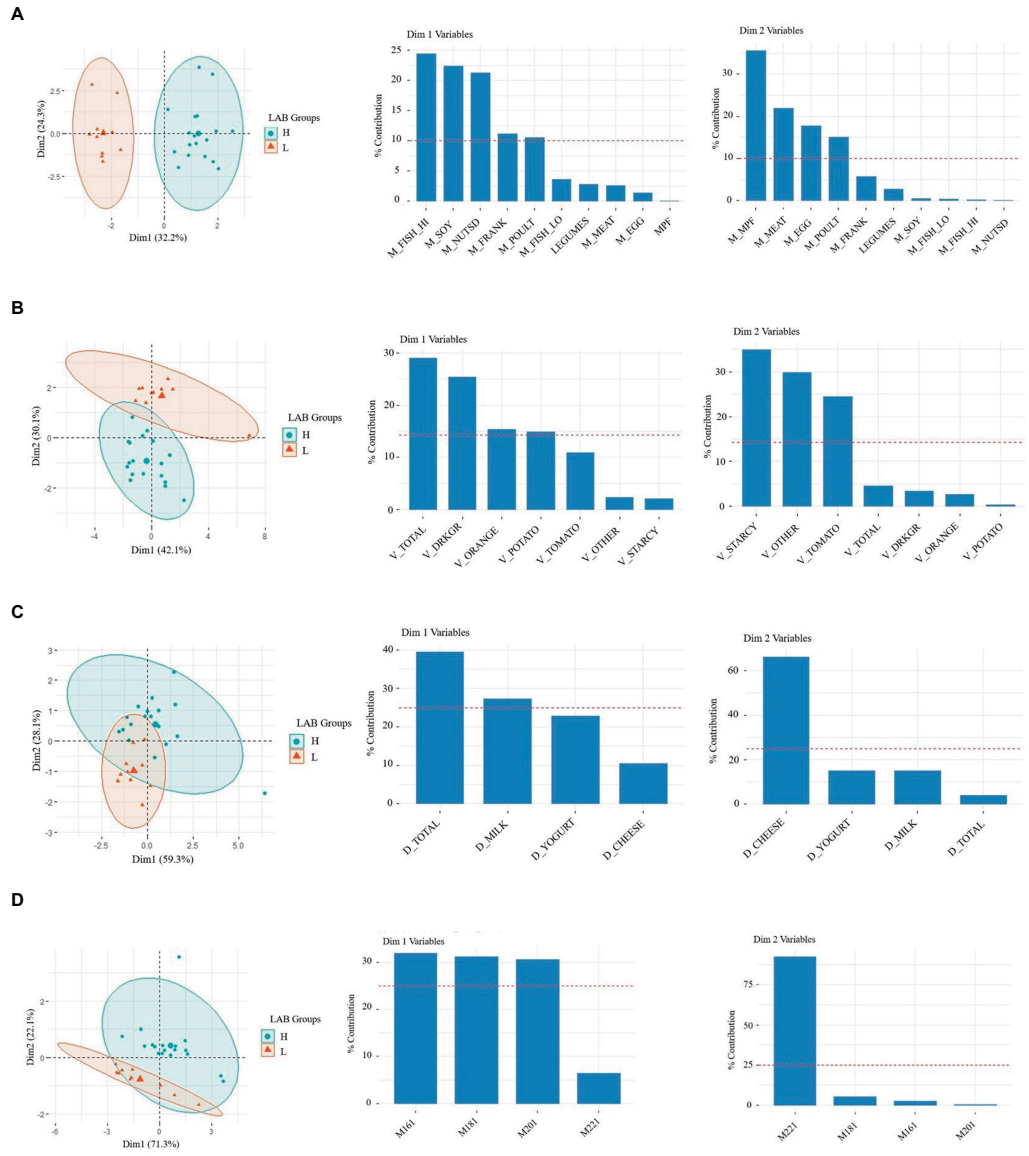
Principal component analysis (PCA) of H-LAB and L-LAB groups by habitual intake of **(A)** meats (oz), **(B)** vegetables (oz), **(C)** dairy (oz), and **(D)** monounsaturated fats (mg). The variables for each of the two dimensions and their % contribution to the variance are given in the eigenvector charts for each PCA plot.

Principal component analysis (PCA) was performed as an exploratory method to visualize differences in the diet of L-LAB and H-LAB individuals. Models were trained with the entire nutrition dataset and with subsets of nutrients defined in Supplementary Table S1. First, the nutrition intake of subjects in the two intervention groups was evaluated. PCA of nutrition intake data where individuals were labeled by LAB counts, H-LAB, and L-LAB were performed next. PCA models obtained from groups for proteins, vegetables, dairy, and monounsaturated fats showed visible separation of H-LAB and L-LAB groups (Figure 1). Within proteins, the intake of fish, soy, nuts, franks, and poultry explained the variance between the two LAB groups (Figure 1A). Similarly, the nutrients that significantly contributed to the separation of the H-LAB and L-LAB individuals in the vegetables PCA were starchy vegetables, other vegetables, and tomatoes (Figure 1B), cheese in the dairy food group PCA (Figure 1C), and erucic acid (C22:1) in the monounsaturated fats group (Figure 1D). These results suggest that intake of a defined set of nutrients could be associated to the LAB stool count of individuals.

### Intake of meats and vegetables are correlated with LAB titers

Given the results obtained with PCA, the nutrition data were then further investigated to identify correlations between LAB CFUs and dietary intake. To first determine differences in habitual intake between the two groups, single factor ANOVA was performed. After multiple test corrections for the 97 different variables included in the analyses (Supplementary Tables S1–S3), it was found that the food groups consumed in significantly higher amounts by the H-LAB group were soy (*p* = 6e-05), cheese (*p* = 1e-05), nuts and seeds (*p* = 8.5e-05), franks (*p* = 0.04), and fish high in N-3 fatty acids (*p* = 1.9e-07). On the contrary, dietary variables consumed in higher amounts by the L-LAB group are starchy vegetables (*p* = 0.003) and tomatoes (*p* = 3.6e-07; Table 2). In agreement with this observation, the L-LAB group had a higher intake of added vitamin E (*p* = 0.0002), and lycopene (*p* = 9.2e-05; Supplementary Tables S2, S3). While the total intake of fats was not significantly different between the groups, it was found that the H-LAB group consumed significantly higher amounts of the saturated fatty acids caprylic acid (C8:0; *p* = 2.4e-06), capric acid (C10:0; *p* = 0.004), lauric acid (C12:0; *p* = 0.001), and myristic acid (C14:0; *p* = 0.009), as well as the monounsaturated acid erucic acid (C22:1; *p* = 0.0001) and the polyunsaturated acid stearidonic acid (C18:4; *p* = 2.7e-06). To confirm the relationship between these nutrient variables and LAB CFUs, linear regression was performed. A positive correlation was observed between the intake of cheese, soy, nuts and seeds, fish high in N-3 fatty acids, and other vegetables, while a negative correlation was observed for tomatoes and lycopene (Table 3).

**Table 2:** Habitual intake represented as adjusted average intake of all measured fatty acids, mean (SD). *p < 0.05.

Fats & fatty acids	Intervention		LAB (CFU)	
	*Placebo*	*L. johnsonii N6.2*	*High*	*Low*
	*n* = 18	*n* = 20	*n* = 18	*n* = 10
Total fat (g)	81.79 ± 14.01	73.68 ± 13.82	88.46 ± 25.41	68.23 ± 13.92
Saturate fats (g)	26.88 ± 4.16	23.99 ± 4.67	29.92 ± 8.15	21.71 ± 2.67
Butryric acid (g)	0.62 ± 0.11	0.54 ± 0.11	0.75 ± 0.24	0.49 ± 0.04
Caproic acid (g)	0.31 ± 0.06	0.28 ± 0.05	0.39 ± 0.13	0.27 ± 0.02
Caprylic acid (g)	0.29 ± 0.06	0.28 ± 0.06	0.38 ± 0.06*	0.21 ± 0.03
Capric acid (g)	0.54 ± 0.12	0.46 ± 0.10	0.66 ± 0.17*	0.38 ± 0.04
Lauric acid (g)	0.87 ± 0.19	1.04 ± 0.33	1.31 ± 0.30*	0.69 ± 0.25
Myristic acid (g)	2.34 ± 0.29	2.12 ± 0.44	2.82 ± 0.67*	1.79 ± 0.19
Palmitic acid (g)	14.36 ± 2.45	12.7 ± 2.61	15.55 ± 4.43	11.94 ± 1.87
Stearic acid (g)	6.70 ± 1.26	5.84 ± 1.10	7.13 ± 2.09	5.29 ± 0.79
Monounsaturated fatty acids (g)	561.51 ± 5.86	27.81 ± 5.32	33.27 ± 9.49	25.24 ± 7.86
Palmitoleic acid (g)	1.26 ± 0.27	1.14 ± 0.26	1.35 ± 0.35	1.07 ± 0.34
Oleic acid (g)	29.29 ± 5.49	26.09 ± 4.93	31.28 ± 8.97	23.64 ± 7.30
Eicosenoic acid (g)	0.21 ± 0.03	0.21 ± 0.03	0.22 ± 0.04	0.18 ± 0.05
Erucic acid (g)	0.02 ± 0.01	0.03 ± 0.01	0.03 ± 0.01*	0.01 ± 0.001
Polyunsaturated fats (g)	16.86 ± 2.82	15.60 ± 2.04	17.95 ± 4.40	15.62 ± 1.90
Linoleic acid (g)	14.74 ± 2.28	13.75 ± 1.75	15.64 ± 3.73	13.81 ± 1.76
γ-Linolenic acid (g)	1.61 ± 0.38	1.34 ± 0.25	1.74 ± 0.39	1.40 ± 0.09
Stearidonic acid (g)	0.02 ± 0.01	0.02 ± 0.004	0.02 ± 0.004*	0.01 ± 0.001
Eicoatetraenoic acid (g)	0.14 ± 0.04	0.15 ± 0.06	0.16 ± 0.07	0.15 ± 0.06
Timnodonic acid (g)	0.06 ± 0.01	0.04 ± 0.01	0.05 ± 0.01	0.03 ± 0.01
DPA (g)	0.02 ± 0.01*	0.02 ± 0.002	0.02 ± 0.004	0.02 ± 0.004
DHA (g)	0.10 ± 0.03	0.08 ± 0.01	0.09 ± 0.02	0.08 ± 0.02

**Table 3:** Linear Regression of adjusted average intake and average log (CFU/g wet stool) LAB.

Dietary variable	Beta coefficient	*R* ^2^	*p*-val
Tomato	−0.056	0.4569	0.008
Other vegetables	0.1503	0.4167	0.02
Cheese	0.1539	0.3789	0.04
Fish_hi	0.0767	0.4116	0.02
Soy	0.0807	0.4767	0.005
Nuts & Seeds	0.2687	0.5102	0.002
Lycopene	−1130.7	0.3741	0.048

Finally, a principal component regression was performed using the previously described PCs and log (CFU/g wet stool) LAB. Regression of the principal components confirmed the results obtained, with the most significant result being that of dimension 2 (Dim2), the second principal component, of the vegetable model which is negatively correlated to CFU LAB (*R*^2^ = 0.52, *p* = 1.35e-05; Supplementary Figure S2A). Additionally, proteins dimension 1 (Dim1) showed significant positive correlation to log CFU’s LAB (*R*^2^ = 0.59, *p* = 1.73e-06; Supplementary Figure S2B). Taken together, the variables of the second dimension of the vegetable model including starchy vegetables, and tomatoes are negatively correlated to the abundancy of LAB. Additionally, the variables of dimension one of the protein model including fish high in N3 fatty acids, soy, nuts and seeds, fermented meats, and poultry have a positive correlation.

### Erucic acid is a predictor of LAB categorization

**Figure 2:**
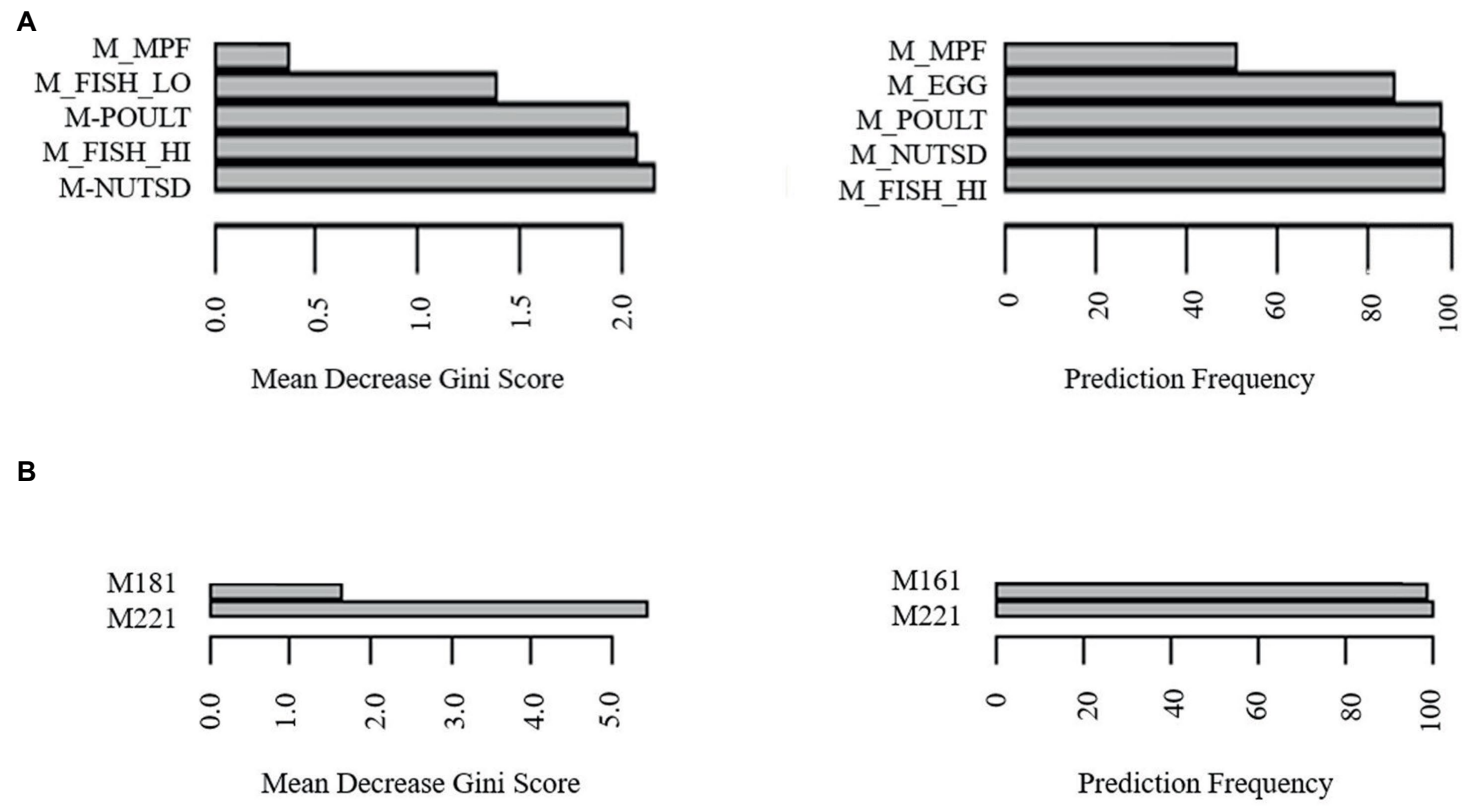
Identification of biomarkers for LAB abundancy by most frequently predictive and greatest importance dietary intake variables of random forest models trained with **(A)** meats, **(B)** monounsaturated fatty acids erucic acid (“M221,” C22:1) and oleic acid (“M181,” C18:1).

To determine potential biomarkers of LAB in the gut of healthy adults, the machine learning techniques extreme gradient boosting and random forest were employed. Extreme gradient boosting was used to build several highly accurate models for the prediction of H-LAB and L-LAB categorization. The most accurate models include those trained with all food groups, proteins, fatty acids (sfats, mfats, and pfats), and vegetables (Table 4). Each separate model had a 100% accuracy and singular variables with 100% importance in the model. The important features of each model were tomato, fish high in M3 fatty acids (M_fish_hi), S080, M221, P184, and tomato, respectively. To validate the results of the extreme gradient boosting decision tree modeling, random forest models were trained with the dietary intake variables and refined over 500 iterations using an 80/20 cross-validation scheme (Table 5). Models with >85% prediction accuracy were considered significant, and variables with both high importance and high frequency of prediction were chosen as potential biomarkers of LAB. First, all nutrition variables were analyzed together to create a predictive model. This model, along with all others, was repeated over 500 iterations to determine its reproducibility. The model with all 97 variables was not accurate in its predictions with highly variable accuracy of 93.8 ± 26% (Table 4). Next, dietary intake variables were then separated into categories (given in Supplementary Table S1). Of these, only two accurate models were found: proteins and monounsaturated fatty acids. The model of protein intake was both accurate and predictive of LAB grouping (accuracy = 98.6 ± 11.8%), with predictor variables being fish high in n-3 fatty acids (“M_FISH_HI”) and nuts and seeds (“M_NUTSD”; Figure 2A). All other food groups were not predictive of LAB group (Table 4). Interestingly, the monounsaturated fatty acid model trained with all monounsaturated fatty acids has a high error rate (accuracy = 87.2 ± 33.4%; Figure 2B). However, the model trained with only erucic acid (“M221,” C22:1) yielded an accuracy of 100%, meaning intake of M221 alone can be used to accurately predict LAB grouping (Figure 2B, Table 4). From these models nuts and seeds, fish high in N-3 fatty acids, and erucic acid (C22:1) were identified as potential biomarkers of LAB.

**Table 4:** Extreme gradient boosting model prediction accuracy (%), *p*-values, and predictors within the model.

Group	Prediction accuracy (%)	*p*-val	Predictor(s)
Food groups	100	0.02328	Tomato
Dairy	87.5	0.3671	Cheese
Proteins	100	0.02328	M_fish_hi
Small nutrients	87.5	0.3671	Alc
Vitamins	87.5	0.03516	Lyco
Sfats	100	0.02328	S080
Mfats	100	0.02328	M221
Pfats	100	0.02328	P184
All	87.5	0.3671	Alc
Fruits	87.5	0.03516	F_other, F_total, F_citmlb
Grains	87.5	0.3671	G_total, G_nwhl
Vegetables	100	0.02328	Tomato
Macros	75	0.9327	Kcal, Carb, Prot, Tfat
Macros + FA	100	0.02328	S080
Fatty acids	100	0.02328	S080
Unsaturated fatty acids	100	0.02328	M221
Minerals	87.5	0.03516	Calc, Iron, Phos, Copp, Sodi

**Table 5:** Random forest prediction accuracy.

Group	Prediction accuracy (%)
All foods	96 ± 24.1
Dairy	57.2 ± 49.5
Proteins	98.6 ± 11.8
Small nutrients	83.4 ± 37.2
Vitamins	43.4 ± 49.6
Saturated fatty acids	34.2 ± 47.5
Polyunsaturated fatty acids	67.8 ± 46.8
Monounsaturated fatty acids	87.2 ± 33.4
Erucic acid	100 ± 0
All variables	93.8 ± 24.1
Fruits	1.2 ± 10.9
Grains	7.2 ± 25.9
Vegetables	76.8 ± 42.3
Macronutrients	3.6 ± 18.6
Macronutrients and fatty acids	65.6 ± 47.6
Fatty acids	73.2 ± 44.3
Unsaturated fatty acids	87.4 ± 33.2
Minerals	0.2 ± 4.5

### Several metabolite concentrations are modified between H-LAB and L-LAB groups

Untargeted metabolomic profiling was performed to evaluate whether nutrient intake maybe reflected in metabolite changes. The fluctuations in metabolites were analyzed by PCA and expressed as fold-change. Similar to the nutrition analyses, it was found that comparing data by intervention group (Ljo versus placebo) did not result in any significant differences between metabolite levels (Fig. S3A-B). Next, the data were stratified by H-and L-LAB groups. While PCA of the metabolite profiles showed only a slight separation between H-LAB and L-LAB groups (Fig. S3C), volcano plot analysis shows four significant features (Supplementary Figure S3D, Table S4). Based on their mass and the Human Metabolome Database (25), these features were putatively identified as chondroitin sulfate E (mass = 134.072, *p* = 0.01) and pyrazine (mass = 81.0448) which were higher in the H-LAB group, while polypropylene glycol (mass = 152.1283 *p* = 0.09) and caproic acid (mass = 117.0912, *p* = 0.08) were higher in the L-LAB group (Supplementary Table S5). When analyzed by timepoint (T), several more significant features were identified with 6 significant features at T1 (Initial sample, no intervention), 16 at T2 (2 weeks into the intervention phase), 15 at T3 (4 weeks into the intervention phase), 53 at T4 (8 weeks into the intervention phase), and 17 at T5 (12 weeks, end point; Supplementary Figures S3E–N). These metabolites were also identified by their mass and are listed in Supplementary Table S4. The relationship between metabolites, dietary intake, and their effect on the presence of LAB is overtly complex and will require further analyses.

### Various species of *Lactobacillus* utilize erucic acid regardless of their mode of fermentation

**Figure 3:**
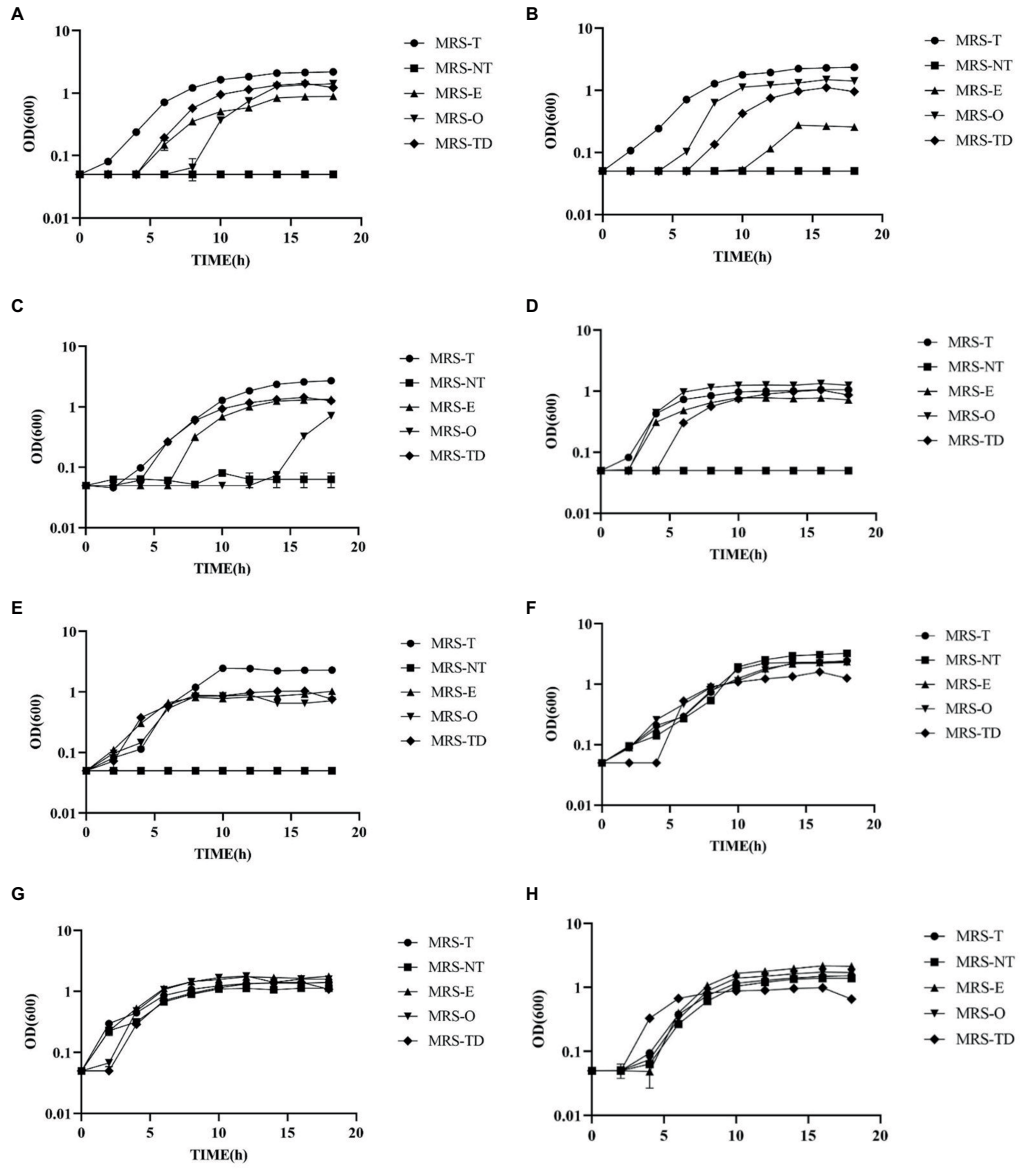
*Lactobacillus* species showed a visible ability to utilize erucic and oleic acid as a sole source of fatty acids. Growth curves of **(A)** *Lactobacillus johnsonii* N6.2, **(B)** *L. johnsonii* ATCC 33200, **(C)** *Lactobacillus amylovorus* ATCC 33620, **(D)** *Ligilactobacillus murinus* 35,020, **(E)** *Limosilactobacillus reuteri* TD1 **(F)** *Ligilactobacillus fermentum* 14,391 **(G)** *Lactipantibacillus plantarum* 25,302, and **(H)** *Lacticaseibacillus casei* ATCC 334, were grown in MRS-NT supplemented with 0.1% oleic acid (MRS-O), 0.1% erucic acid (MRS-E), Tween 80 (MRS-T), without Tween 80 (MRS-NT), and with Tween 80 and 0.1% DMSO (MRS-TD).

The analysis of habitual diet indicated a relationship between the intake of monounsaturated fatty acids erucic acid (C22:1) and oleic acid (C18:1) and LAB counts. To further investigate these results, *in vitro* growth kinetic experiments were performed with several species formerly classified as of *Lactobacillus* to determine their ability to utilize the alternate fatty acid erucic acid opposed to Tween 80 as its fatty acid source. The strains (listed in Supplementary Table S5) were each grown in triplicate in MRS-E, MRS-O, MRS-T, MRS-NT, and MRS-TD (MRS-T with DMSO 0.1%, vehicle control) for a total of 18 h. Growth kinetics μmax and doubling time (DT) were calculated for each culture. Strains were chosen based on their modes of fermentation to compare strains of various metabolic capabilities as follows: homofermentative (*L. johnsonii* N6.2, *L. johnsonii* ATCC 33200, *Lactobacillus amylovorus* ATCC 33620, *Ligilactobacillus murinus* ATCC 35020; heterofermentative (*Limosilactobacillus fermentum* ATCC14391, *Limosilactobacillus Reuteri* TD1), and facultatively heterofermentative (*Lactipantibacillus plantarum* ATCC25302, and *Lacticaseibacillus casei* ATCC 334; Supplementary Table S5). While most of the *Lactobacillus* strains are auxotrophic for fatty acids and require the addition of Tween 80 to the media, three of the selected strains, *L. plantarum* ATCC25302, *L. casei* ATCC 334, and *L. fermentum* ATCC14391, have the genes that encode for the necessary enzymes of the FASII pathway required for *de novo* biosynthesis of fatty acids (Supplementary Table S5). As expected, these three strains were able to grow at comparable speed in all culture media (Figure 3).

It was found that all the homofermentative *Lactobacillus* species required fatty acids for growth as it is evidenced by the absence of growth in MRS-NT. Interestingly, all the strains tested were able to grow in presence of erucic acid albeit with significant differences. *Lactobacillus johnsonii* N6.2 was able to utilize Tween 80 (MRS-T), erucic acid (MRS-E), and oleic acid (MRS-O) as well as the DMSO vehicle control (MRS-TD). The extended lag phase observed in MRS-E seems to be due to the DMSO vehicle as the growth patterns are visually similar (Figure 3A). However, there is a significant decrease in μ-max and increase in DT in cells grown in MRS-E as compared to MRS-O, MRS-T, and MRS-TD (Supplementary Figure S5). Cells grown in MRS-O have an 8 h lag as well as significantly lower μ-max compared to MRS-T, but not MRS-TD. Cells in MRS-O also had lower DT than any other condition (Supplementary Figure S5). *Lactobacillus johnsonii* ATCC 33200 also utilized both oleic and erucic acid, though in MRS-E, there was a 10 h lag phase (Figure 3B). Cells grown in MRS-O had significantly higher μ-max compared to each condition and lower DT compared to the vehicle control MRS-TD (Supplementary Figure S5). *Lactobacillus amylovorus* ATCC 33620 had no detectible growth in MRS-O until 14 h incubation time (Figure 3C). In MRS-E, there were no significant differences in μ-max compared to MRS-TD but had significantly longer DT compared to MRS-DT (Supplementary Figure S5). *Ligilactobacillus murinus* ATCC 35020 had measurable growth in all conditions, with higher μ-max in MRS-O and lower DT compared to MRS-E (Figure 3D, Supplementary Figure S5).

Within the heterofermentative *L. reuteri* TD1 had no significant differences in μmax between MRS-E and MRS-O, however in MRS-E the DT was significantly lower (Figure 3E). *Ligilactobacillus fermentum* 14391 which encodes the necessary enzymes of the FASII pathway, had growth in MRS-NT as expected. It also had no difference in μ-max comparing MRS-O and MRS-E but had significantly lower DT in MRS-O compared to MRS-E (Figure 3F). The facultative heterofermentative strains *L. plantarum* ATCC 81 and *L. casei* ATCC 334 both encode the necessary enzymes of the FASII pathway and thus, as expected, both were able to grow in MRS-NT (Figures 3G,H). *Lactipantibacillus plantarum* ATCC 25302 had lower μ-max in MRS-E compared to MRS-O, and lower DT in MRS-O compared to MRS-E. *Lacticaseibacillus casei* ATCC 334 had lower μ-max and DT in MRS-O compared to MRS-E (Supplementary Figure S5). Together, these results indicate that the use of erucic acid by some strains of lactic acid bacteria may allow for a competitive advantage in the human gut and may be used as a natural dietary prebiotic to consider during probiotic interventions.

## Discussion

This study aimed to evaluate the use of dietary intake data to establish an accurate and reproducible method of predicting the presence of specific bacteria or, in this case, the group of lactic acid bacteria in the human gut. Using the combined analyses of LAB counts and habitual diet using machine learning techniques, it was possible to determine that specific dietary components are key drivers in the colonization of lactic acid bacteria in human subjects. The identification of dietary patterns conducive to the presence of LAB in the human gut can be utilized in future interventions during the screening phase to prospective evaluate the influence of the residing microbiota on the outcomes of probiotic interventions.

The global relationship between diet and the microbiome has been explored in late years. The influence of type of diet has been studied on health outcomes such as immunological tolerance and gut permeability (3–5). Metabolomics analysis in addition to diet and the microbiome has been used recently to further understand the mechanism of these outcomes in the gastrointestinal microbiome. For example, using multi-omics analysis approach, a positive correlation has been found between habitual intake of vitamin E, folate, cheese, lutein and zeaxanthin, tomatoes, and the metabolite hexadecanedioate and *Bifidobacterium* (26). In our study, while metabolite profiles did not result in significant discrimination between the L-LAB and H-LAB individuals nor any significant correlations to CFU’s LAB, several up-and down-regulated metabolites were identified at each of the five timepoints within the study. These metabolites were not correlated to any dietary intake variables, and further analysis will be required to elucidate any direct relationship between diet, metabolites, and LAB counts.

The results of our previous study indicated the effect of LAB content in the gut microbiota on the outcomes of treatment with *L. johnsonii* N6.2 as significant changes in the IDO pathway were only observed when individuals were stratified by LAB groups (10). The analyses of dietary intake based on intervention groups found no significant differences among the groups; however, significant differences were observed regarding the LAB abundancy groups. First, a negative correlation was observed between consumption of tomato and LAB colonization of the gut. These results are in agreement with a recent study in which a variety of dietary patterns scoring systems were used to perform correlations with the fecal microbiome. The authors reported that the Alternative Healthy Eating Index (AHEI) and Modified Mediterranean Diet were negatively associated to fecal *Lactobacillus* levels (27). These scoring systems are in part characterized by high intake of vegetables (>5 servings per day) (28, 29). However, specific components were not directly associated to *Lactobacillus* abundancy.

In our study, we also identified potential biomarkers of LAB. Among these possible biomarkers for H-LAB were intake of cheese, nuts and seeds, fish high in N-3 fatty acids, and erucic acid (M221). Intake of erucic acid, specifically, was identified as a strong predictor of LAB categorization. Erucic acid is an important ligand of the transcription factor PPAR-δ and is a precursor of nervonic acid, a component of myelin, and is currently utilized as a treatment for adrenoleukodystrophy (30). It has also been investigated for therapeutic properties in multiple sclerosis, Alzheimer’s disease, and Huntington’s disease (31–33). In the human diet, sources of erucic acid include canola/rapeseed oils, mustard and mustard oil, broccoli, cod, salmon, mackerel, and herring (34–39). While the specific sources of erucic acid in the ASA24 questionnaires are not specified, the potential sources of erucic acid measured in this study would be oils, nuts and seeds, and fish high in N-3 fatty acids.

In this study, individuals in the H-LAB group consumed a predicted average of 23.9 mg/day erucic acid. The role of lipids as drivers of bacterial colonization has had little exploration. It has been reported that fatty acids can modulate the abundancy of *Lactobacillus* sp. depending on the source and the degree of unsaturation of the lipids. For example, the presence of high concentrations of saturated fats, such as lard in the diet, lead to decreases in *Lactobacillus* sp., while unsaturated fats such as those sourced from fish, result in increases (12, 40–44). While unsaturated fats have been previously shown to increase *Lactobacillus* sp., the predictive relationship between erucic acid (C22:1) and LAB is a novel finding of this study (12). Furthermore, we were able to determine that *L. johnsonii* N6.2 as well as other homofermentative *Lactobacillus* species that are auxotrophic fatty acids, can utilize erucic acid as the sole source of fatty acid.

The use of multivariate statistical analysis enabled the elucidation of the interaction between dietary variables, their derived metabolites, and their combined influence on the gut microbiota. This method has potential to be further applied to anticipate the success, colonization, and beneficial impacts in individuals undergoing microbiome-based therapies based on their diet as a form of personalized medicine. As probiotics become more widely used as biologics, diet should be considered a confounding factor in studies regarding clinical outcomes and during evaluations of responders versus non-responders to microbiome-centric interventions.

## Data availability statement

The original contributions presented in the study are included in the article/Supplementary material, further inquiries can be directed to the corresponding author.

## Ethics statement

This study was carried out in accordance with the recommendations of the Institutional Review Board (# 201400370) at the University of Florida with written informed consent from all subjects. All subjects gave written informed consent in accordance with the Declaration of Helsinki. The protocol was approved by the Institutional Review Board at University of Florida (this trial was registered at http://clinicaltrials.gov as NCT02349360). The patients/participants provided their written informed consent to participate in this study.

## Author contributions

ST, TG, and AF performed the experiments. CG, WD, and GL contributed to the conception and design of the study. ST, EM, LS, and AC analyzed the data. ST and GL wrote the original draft. AC, WD, CG, and GL reviewed and edited the manuscript. All authors contributed to the article and approved the submitted version.

## Funding

This study was funded by the National Institute of Diabetes and Digestive and Kidney Diseases of the National Institutes of Health under award number R01DK121130. The content is solely the responsibility of the authors and does not necessarily represent the official views of the National Institutes of Health.

## Conflict of interest

GL holds U.S. patent No. 9,474,773 and 9,987,313 on *Lactobacillus johnsonii* N6.2.

The remaining authors declare that the research was conducted in the absence of any commercial or financial relationships that could be constructed as a potential conflict of interest.

## Publisher’s note

All claims expressed in this article are solely those of the authors and do not necessarily represent those of their affiliated organizations, or those of the publisher, the editors and the reviewers. Any product that may be evaluated in this article, or claim that may be made by its manufacturer, is not guaranteed or endorsed by the publisher.
