# Supplementary material for: Identification of food and nutrient components as predictors of Lactobacillus colonization
Source: Front Nutr. 2023 Apr 21;10:1118679. doi: 10.3389/fnut.2023.1118679 (PMC10160632; doi:10.3389/fnut.2023.1118679)
Supplement: Supplementary file 1 [file Table_1.DOCX]

Table S1. All dietary variables measured by ASA24

| **Macronutrients** | **Vitamins** | **Minerals** | **Fruits** |
| --- | --- | --- | --- |
| Protein | Vitamin A (mcg) | Calcium (mg) | Total fruits (cup) |
| Carbohydrates | Retinol (mcg) | Iron (mg) | Citrus, melons, berries (cup) |
| Sugars | Carotinoids | Magnesium (mg) | Other fruits (cup) |
| Fiber | α-carotene (mcg) | Phosphorus (mg) | Whole fruits (cup) |
| Total Fat | β-carotene (mcg) | Potassium (mg) | **Proteins** |
| Saturate fats (g) | β-cryptoxanthin (mcg) | Sodium (mg) | Meat, poultry, fish (oz) |
| Butryric acid (g) | Lutein + zeaxanthin (mcg) | Zinc (mg) | Meat (oz) |
| Caproic acid (g) | Lycopene (mcg) | Copper (mg) | Franks, sausages, lunch meats (oz) |
| Caprylic acid (g) | Thiamin (mg) | Selenium (mcg) | Poultry (oz) |
| Capric acid (g) | Riboflavin (mg) | **Other** | Fish high in n-3 FA (oz) |
| Lauric acid (g) | Niacin (mg) | Caffeine (mg) | Fish low in n-3 FA (oz) |
| Myristic acid (g) | Vitamin B6 (mg) | Theobromine (mg) | Eggs (oz) |
| Palmitic acid (g) | Vitamin B12 (mcg) | Water (g) | Legumes (oz) |
| Stearic acid (g) | Vitamin B12, added (mcg) | Alcohol (g) | Soy (oz) |
| Monounsaturated fatty acids (g) | Folate, total (mcg) | **Grains** | Nuts and seeds (oz) |
| Palmitoleic acid (g) | Folate, DFE (mcg) | Total grains (oz) | **Dairy** |
| Oleic acid (g) | Folic acid (mcg) | Whole grains (oz) | Total dairy (cup) |
| Eicosenoic acid (g) | Folate, food (mcg) | Non-whole grains (oz) | Milk (cup) |
| Erucic acid (g) | Vitamin C (mg) | **Vegetables** | Yogurt (cup) |
| Polyunsaturated fats (g) | Vitamin E (mg) | Total vegetables (cup) | Cheese (cup) |
| Linoleic acid (g) | Vitamin E, added (mg) | Dark-green vegetables (cup | **Extras** |
| γ-Linolenic acid (g) | Vitamin K (mcg) | Orange vegetables | Discretionary Oils (g) |
| Stearidonic acid (g) | Vitamin D | White potatoes (cup) | Discretionary Solid fats (g) |
| Eicoatetraenoic acid (g) | Choline (mg) | Other starchy vegetables (cup) | Added sugars (tsp) |
| Timnodonic acid (g) |  | Tomatoes (cup) | Alcoholic beverages (total drinks) |
| DPA (g) |  | Other vegetables (cup) |  |
| DHA (g) |  |  |  |
|  |  |  |  |
|  |  |  |  |
