# Supplementary material for: Identification of food and nutrient components as predictors of Lactobacillus colonization
Source: Front Nutr. 2023 Apr 21;10:1118679. doi: 10.3389/fnut.2023.1118679 (PMC10160632; doi:10.3389/fnut.2023.1118679)
Supplement: Supplementary file 2 [file Table_2.DOCX]

Table S2. Habitual intake represented as adjusted average intake of all measured food groups, mean (SD). *p<0.05.

| Food Group | Intervention | | LAB (CFU) |  |
| --- | --- | --- | --- | --- |
|  | *Placebo* | *L. johnsonii N6.2* | *High* | *Low* |
|  | n= 18 | n= 20 | n=18 | n=10 |
| Total grains (oz) | 6.65 ± 1.28* | 5.27 ± 0.42 | 6.55 ± 1.06 | 5.66 ± 0.29 |
| Whole grains (oz) | 0.95 ± 0.40* | 0.47 ± 0.18 | 0.79 ± 0.38 | 0.62 ± 0.45 |
| Non-whole grains (oz) | 5.70 ± 1.25 | 4.79 ± 0.43 | 5.75 ± 1.13 | 5.04 ± 0.45 |
| Total vegetables (cup) | 1.76 ± 0.34 | 1.61 ± 0.71 | 1.79 ± 0.37 | 1.75 ± 0.86 |
| Dark-green vegetables (cup | 0.27 ± 0.11 | 0.26 ± 0.21 | 0.27 ± 0.11 | 0.29 ± 0.17 |
| Orange vegetables | 0.10 ± 0.08 | 0.10 ± 0.04 | 0.07 ± 0.03 | 0.09 ± 0.02 |
| White potatoes (cup) | 0.21 ± 0.05 | 0.31 ± 0.21 | 0.28 ± 0.05 | 0.34 ± 0.29 |
| Starchy vegetables (cup) | 0.08 ± 0.02 | 0.08 ± 0.01 | 0.08 ± 0.02 | 0.11 ± 0.01* |
| Tomatoes (cup) | 0.40 ± 0.07 | 0.37 ± 0.06 | 0.35 ± 0.04 | 0.49 ± 0.04* |
| Other vegetables (cup) | 0.69 ± 0.24 | 0.48 ± 0.13 | 0.74 ± 0.22 | 0.42 ± 0.08 |
| Total fruits (cup) | 1.19 ± 0.90 | 0.81 ± 0.24 | 1.42 ± 1.10 | 0.95 ± 0.25 |
| Citrus, melons, berries (cup) | 0.59 ± 0.74 | 0.22 ± 0.07 | 0.58 ± 0.88 | 0.27 ± 0.05 |
| Other fruits (cup) | 0.61 ± 0.23 | 0.58 ± 0.21 | 0.84 ± 0.34 | 0.68 ± 0.33 |
| Whole fruits (cup) | 0.86 ± 0.37 | 0.66 ± 0.20 | 0.96 ± 0.33 | 0.82 ± 0.28 |
| Total dairy (cup) | 1.48 ± 0.25 | 1.31 ± 0.28 | 1.68 ± 0.50 | 1.25 ± 0.14 |
| Milk (cup) | 0.53 ± 0.20 | 0.5 ± 0.16 | 0.63 ± 0.35 | 0.64 ± 0.09 |
| Yogurt (cup) | 0.08 ± 0.08 | 0.12 ± 0.11 | 0.14 ± 0.14 | 0.12 ± 0.11 |
| Cheese (cup) | 0.86 ± 0.14 | 0.69 ± 0.20 | 0.91 ± 0.15* | 0.49 ± 0.14 |
| Meat, poultry, fish (oz) | 4.22 ± 1.34 | 4.13 ± 0.74 | 4.06 ± 1.38 | 4.08 ± 1.09 |
| Meat (oz) | 1.43 ± 0.40 | 1.55 ± 0.40 | 1.46 ± 0.51 | 1.15 ± 0.42 |
| Franks, sausages, lunch meats (oz) | 0.68 ± 0.45 | 0.38 ± 0.11 | 0.55 ± 0.18* | 0.30 ± 0.05 |
| Poultry (oz) | 1.61 ± 0.48 | 1.79 ± 0.24 | 1.57 ± 0.39 | 2.27 ± 0.64 |
| Fish high in n-3 FA (oz) | 0.20 ± 0.08 | 0.20 ± 0.07 | 0.24 ± 0.07* | 0.02 ± 0.01 |
| Fish low in n-3 FA (oz) | 0.32 ± 0.12 | 0.22 ± 0.09 | 0.28 ± 0.10 | 0.33 ± 0.12 |
| Eggs (oz) | 0.54 ± 0.23 | 0.72 ± 0.71 | 0.82 ± 0.55 | 0.66 ± 0.75 |
| Legumes (oz) | 0.25 ± 0.15* | 0.10 ± 0.07 | 0.14 ± 0.09 | 0.20 ± 0.07 |
| Soy (oz) | 0.20 ± 0.12* | 0.07 ± 0.03 | 0.23 ± 0.09* | 0.04 ± 0.01 |
| Nuts and seeds (oz) | 1.00 ± 0.27 | 0.97 ± 0.60 | 0.95 ± 0.27* | 0.39 ± 0.04 |
| Discretionary Oils (g) | 19.55 ± 2.44 | 16.78 ± 2.87 | 20.52 ± 3.13 | 15.00 ± 5.57 |
| Discretionary Solid fats (g) | 43.31 ± 10.23 | 37.75 ± 6.39 | 49.25 ± 13.98 | 35.56 ± 3.50 |
| Added sugars (tsp) | 11.03 ± 2.60 | 9.20 ± 1.89 | 11.08 ± 3.81 | 8.75 ± 1.24 |
| Alcoholic beverages (total drinks) | 0.27 ± 0.12 | 0.23 ± 0.13 | 0.22 ± 0.16 | 0.05 ± 0.02 |
