# Supplementary material for: Identification of food and nutrient components as predictors of Lactobacillus colonization
Source: Front Nutr. 2023 Apr 21;10:1118679. doi: 10.3389/fnut.2023.1118679 (PMC10160632; doi:10.3389/fnut.2023.1118679)
Supplement: Supplementary file 3 [file Table_3.DOCX]

Table S3. Habitual intake represented as adjusted average intake of all measured nutrients, mean (SD). *p<0.05.

| Nutrients | Intervention | | LAB (CFU) |  |
| --- | --- | --- | --- | --- |
|  | *Placebo* | *L. johnsonii N6.2* | *High* | *Low* |
|  | n= 18 | n= 20 | n=18 | n=10 |
| Protein (g) | 84.09 ± 35.33 | 157.73 ± 141.82 | 84.24 ± 21.32 | 74.75 ± 12.28 |
| Carbohydrates (g) | 232.43 ± 64.14 | 194.35 ± 17.09 | 236.37 ± 76.65 | 212.02 ± 13.99 |
| Sugars (g) | 86.95 ± 27.71 | 72.91 ± 6.52 | 94.57 ± 42.97 | 74.50 ± 2.93 |
| Fiber (g) | 20.28 ± 4.62 | 15.70 ± 3.55 | 18.73 ± 5.03 | 18.08 ± 4.85 |
| Cholesterol (mg) | 278.66 ± 79.18 | 320.1 ± 189.27 | 362.11 ± 178.58 | 295.34 ± 177.92 |
| Water (g) | 2521.69 ± 789.73 | 1999.87 ± 377.93 | 2288.91 ± 970.72 | 2380.71 ± 227.54 |
| Alcohol (g) | 3.88 ± 1.59 | 3.27 ± 1.83 | 3.06 ± 2.00 | 0.68 ± 0.4 |
| Vitamin A (mcg) | 724.94 ± 155.71 | 685.59 ± 177.64 | 754.36 ± 222.33 | 730.26 ± 203.30 |
| Retinol (mcg) | 406.40 ± 89.03 | 403.74 ± 71.43 | 468.9 ± 136.80 | 423.21 ± 47.08 |
| α-carotene (mcg) | 570.64 ± 217.24 | 396.90 ± 103.14 | 480.18 ± 207.46 | 491.62 ± 98.08 |
| β-carotene (mcg) | 3474.26 ± 1018.98 | 3155.22 ± 1750.14 | 3131.92 ± 1099.76 | 3401.63 ± 2624.42 |
| β-cryptoxanthin (mcg) | 124.93 ± 37.94* | 48.97 ± 12.68 | 129.20 ± 62.33 | 69.17 ± 19.39 |
| Lutein + zeaxanthin (mcg) | 2725.82 ± 617.21 | 3467.50 ± 3997.85 | 2854.95 ± 667.26 | 3815.76 ± 5873.09 |
| Lycopene (mcg) | 7561.74 ± 2014.23 | 6493.96 ± 878.59 | 6249.01 ± 1355.05 | 9217.6 ± 569.43* |
| Thiamin (mg) | 1.78 ± 0.41* | 1.36 ± 0.10 | 1.75 ± 0.38 | 1.40 ± 0.08 |
| Riboflavin (mg) | 2.00 ± 0.44 | 1.78 ± 0.26 | 2.09 ± 0.64 | 1.87 ± 0.13 |
| Niacin (mg) | 23.91 ± 5.64 | 21.59 ± 2.02 | 22.10 ± 5.76 | 22.90 ± 4.43 |
| Vitamin B6 (mg) | 1.94 ± 0.52 | 1.78 ± 0.27 | 1.86 ± 0.64 | 1.946 ± 0.560 |
| Vitamin B12 (mcg) | 3.97 ± 0.66 | 4.21 ± 0.57 | 4.35 ± 0.78 | 4.006 ± 0.761 |
| Vitamin B12, added (mcg) | 0.74 ± 0.41 | 0.81 ± 0.29 | 0.52 ± 0.24 | 1.028 ± 0.554 |
| Folate, total (mcg) | 473.04 ± 135.84 | 371.52 ± 25.69 | 440.89 ± 124.91 | 394.14 ± 19.339 |
| Folate, DFE (mcg) | 603.14 ± 154.40 | 487.97 ± 30.25 | 554.43 ± 137.80 | 524.05 ± 31.89 |
| Folic acid (mcg) | 186.20 ± 38.15 | 166.20 ± 16.29 | 162.20 ± 19.08 | 185.61 ± 26.97 |
| Folate, food (mcg) | 286.36 ± 110.20 | 205.20 ± 45.11 | 279.65 ± 98.26 | 208.61 ± 32.83 |
| Vitamin C (mg) | 84.77 ± 54.05 | 60.15 ± 23.85 | 90.98 ± 69.01 | 68.43 ± 24.44 |
| Vitamin E (mg) | 8.86 ± 1.14 | 8.16 ± 1.84 | 8.85 ± 1.62 | 8.73 ± 2.11 |
| Vitamin E, added (mg) | 0.34 ± 5.5E-17 | 0.05 ± 0.16* | 0.13 ± 0.06 | 1.11 ± 0.65* |
| Vitamin K (mcg) | 182.77 ± 43.29 | 198.17 ± 171.39 | 191.91 ± 46.10 | 210.94 ± 253.38 |
| Vitamin D | 3.80 ± 0.73 | 3.39 ± 0.23 | 4.16 ± 0.94 | 3.87 ± 0.26 |
| Choline (mg) | 318.38 ± 76.71 | 301.05 ± 116.30 | 348.40 ± 134.62 | 297.88 ± 114.01 |
| Calcium (mg) | 912.41 ± 148.24 | 777.96 ± 107.27 | 976.14 ± 289.66 | 831.80 ± 63.16 |
| Iron (mg) | 15.97 ± 3.22 | 13.30 ± 1.66 | 15.30 ± 3.40 | 14.25 ± 1.51 |
| Magnesium (mg) | 324.81 ± 56.67 | 267.98 ± 34.01 | 317.05 ± 75.89 | 277.12 ± 40.98 |
| Phosphorus (mg) | 1315.41 ± 227.42 | 1162.94 ± 150.46 | 1361.07 ± 333.32 | 1150.43 ± 110.43 |
| Potassium (mg) | 2641.57 ± 714.07 | 2287.40 ± 491.18 | 2762.15 ± 940.73 | 2427.87 ± 603.11 |
| Sodium (mg) | 3579.19 ± 471.25 | 3169.70 ± 444.29 | 3610.04 ± 686.48 | 3351.91 ± 290.56 |
| Zinc (mg) | 11.33 ± 1.30 | 11.34 ± 1.24 | 11.96 ± 1.70 | 11.08 ± 1.69 |
| Copper (mg) | 1.49 ± 0.34 | 1.29 ± 0.18 | 1.50 ± 0.4 | 1.25 ± 0.29 |
| Selenium (mcg) | 112.23 ± 8.21 | 108.95 ± 11.48 | 116.65 ± 23.12 | 101.32 ± 11.14 |
| Caffeine (mg) | 94.09 ± 42.35 | 70.71 ± 23.44 | 90.70 ± 46.20 | 76.32 ± 32.28 |
| Theobromine (mg) | 38.76 ± 14.11 | 26.24 ± 3.81 | 30.71 ± 3.16 | 32.23 ± 13.54 |
