# Supplementary material for: Identification of food and nutrient components as predictors of Lactobacillus colonization
Source: Front Nutr. 2023 Apr 21;10:1118679. doi: 10.3389/fnut.2023.1118679 (PMC10160632; doi:10.3389/fnut.2023.1118679)
Supplement: Supplementary file 4 [file Table_4.DOCX]

Table S4. Significant metabolites from all timepoints and timepoints 1-5 corresponding to pre-intervention, intervention week 2, intervention week 4, intervention week 8, and post-intervention respectively, of individuals with L-LAB and H-LAB. Metabolites were predicted using the mass-to-charge ratios and ionization mode were used to search the Human Metabolite Database (HMDB). Metabolites with values Fold Change (FC) > 1, log_2_(FC) > 0 are significantly higher in H-LAB, and values FC < 1, log_2_(FC) < 0, are significantly higher in L-LAB.

| **All Timepoints** | | | | | |
| --- | --- | --- | --- | --- | --- |
| **ID** | **Mass** | **FC** | **log2(FC)** | **raw.pval** | **-log10(p)** |
| Polypropylene glycol (-) | 152.1283-4.29 | 0.45 | -1.15 | 0.09 | 1.03 |
| Caproic acid (-) | 117.0912-3.48 | 0.50 | -1.01 | 0.08 | 1.08 |
| Pyrazine | 81.0448-4.81 | 2.10 | 1.07 | NA | NA |
| Chondroitin sulfate E (-) | 134.0720-8.76 | 2.87 | 1.52 | 0.01 | 1.84 |

|  |  |  |  |  |  |
| --- | --- | --- | --- | --- | --- |
| **Timepoint 1** | | | | | |
| **ID** | **Mass** | **FC** | **log2(FC)** | **raw.pval** | **-log10(p)** |
| Polypropylene glycol | 117.0912-3.48 | 0.34 | -1.54 | 0.02 | 1.61 |
| N/A(+) | 128.0549-4.26 | 0.48 | -1.07 | 0.05 | 1.32 |
| 4-Hydroxy-3-methoxy-cinnamoylglycine | 252.0862-6.55 | 2.02 | 1.02 | 0.02 | 1.65 |
| 4-Dodecylbenzenesulfonic acid | 349.1812-5.83 | 3.07 | 1.62 | 0.02 | 1.71 |
| Indoleacetic acid (+) | 176.0702-3.73 | 3.72 | 1.90 | 0.02 | 1.78 |
| Gerberinol (+) | 365.1030-1.05 | 4.58 | 2.19 | 0.04 | 1.44 |

|  |  |  |  |  |  |
| --- | --- | --- | --- | --- | --- |
| **Timepoint 2** | | | | | |
| **ID** | **Mass** | **FC** | **log2(FC)** | **raw.pval** | **-log10(p)** |
| N-(1-Deoxy-1-fructosyl)methionine (+) | 294.1022-1.38 | 0.25 | -1.98 | 0.04 | 1.45 |
| N/A(+) | 365.1030-1.05 | 0.36 | -1.48 | 0.01 | 2.19 |
| N/A(+) | 129.0445-4.12 | 0.40 | -1.31 | 0.00 | 2.92 |
| Indoleacrylic acid (+) | 188.0701-7.67 | 0.43 | -1.21 | 0.04 | 1.36 |
| 9-Carboxymethoxymethylguanine (+) | 257.0988-6.24 | 0.44 | -1.18 | 0.03 | 1.52 |
| N/A(+) | 331.0718-5.01 | 0.46 | -1.12 | 0.04 | 1.35 |
| {[3-hydroxy-5-(2-hydroxy-4-imino-1,4-dihydropyrimidin-1-yl)oxolan-2-yl]methoxy}sulfonic acid (+) | 290.0448-5.01 | 0.47 | -1.09 | 0.02 | 1.67 |
| Lamivudine sulfoxide | 246.0541.5.01 | 0.48 | -1.05 | 0.02 | 1.67 |
| Diisopentyl thiomalate (+) | 291.1632-4.50 | 0.48 | -1.04 | 0.03 | 1.58 |
| N-Acetylcadaverine (+) | 145.1329-1.20 | 2.17 | 1.12 | 0.03 | 1.48 |
| Indoxyl (+) | 134.0605-7.14 | 2.26 | 1.17 | 0.02 | 1.64 |
| 3-methyl-2,3-dihydro-1H-pyrrol-2-ol (+) | 100.0755-1.43 | 2.54 | 1.35 | 0.03 | 1.51 |
| Carvone | 133.1024-6.17 | 2.62 | 1.39 | 0.05 | 1.32 |
| N-Acetylleucine (+) | 174.1124-5.39 | 2.69 | 1.43 | 0.04 | 1.37 |
| N/A(+) | 263.1260-8.10 | 3.08 | 1.62 | 0.01 | 2.17 |
| 3-[(3-Methylbutyl)nitrosoamino]-2-butanone (+) | 187.1435-4.84 | 4.46 | 2.16 | 0.02 | 1.78 |

|  |  |  |  |  |  |
| --- | --- | --- | --- | --- | --- |
| **Timepoint 3** | | | | | |
| ID | **Mass** | **FC** | **log2(FC)** | **raw.pval** | **-log10(p)** |
| trans-Cinnamyl alcohol (-) | 135.0807-6.59 | 0.31 | -1.69 | 0.04 | 1.36 |
| N/A(-) | 331.0718-5.01 | 0.38 | -1.41 | 0.02 | 1.76 |
| N/A(-) | 290.0448-5.01 | 0.39 | -1.34 | 0.01 | 1.85 |
| Carvone (-) | 133.1014-9.11 | 0.43 | -1.22 | 0.04 | 1.40 |
| [2-(1,2,3-trihydroxypropoxy)ethoxy]sulfonic acid (-) | 215.0231-5.01 | 0.45 | -1.14 | 0.05 | 1.34 |
| N/A | 257.2231-8.96 | 0.46 | -1.13 | 0.03 | 1.49 |
| N/A(-) | 239.0780-6.78 | 0.47 | -1.10 | 0.01 | 1.84 |
| N/A(-) | 296.9938-6.36 | 2.01 | 1.01 | 0.03 | 1.57 |
| Leucylleucine (-) | 245.1852-5.92 | 2.03 | 1.02 | 0.05 | 1.32 |
| Coumarinic acid (-) | 165.0548-5.83 | 2.06 | 1.04 | 0.02 | 1.64 |
| D-Phenyllactic acid (-) | 184.0970-6.91 | 2.45 | 1.29 | 0.02 | 1.74 |
| N/A(-) | 317.8999-3.94 | 2.89 | 1.53 | 0.04 | 1.41 |
| N/A(-) | 174.1124-5.39 | 3.41 | 1.77 | 0.00 | 2.43 |
| N/A(-) | 317.8988-2.67 | 3.41 | 1.77 | 0.03 | 1.51 |
| N/A(-) | 100.0755-1.43 | 3.71 | 1.89 | 0.03 | 1.52 |

|  |  |  |  |  |  |
| --- | --- | --- | --- | --- | --- |
| **Timepoint 4** | | | | | |
| ID | **Mass** | **FC** | **log2(FC)** | **raw.pval** | **-log10(p)** |
| Isometheptene | 142.1588-4.96 | 0.28 | -1.86 | 0.02 | 1.77 |
| Hypoxanthine | 136.038511 | 0.41 | -1.28 | 0.05 | 1.34 |
| S-aminomethyldihydrolipoamide | 237.1096-8.75 | 0.42 | -1.25 | 0.00 | 2.33 |
| 2,3-Dihydro-5-methyl-1H-pyrrolizine-7-carboxaldehyde | 150.0916-1.82 | 0.44 | -1.20 | 0.03 | 1.59 |
| N/A | 291.1632-4.50 | 0.45 | -1.16 | 0.05 | 1.32 |
| Pyrimethanil | 200.1181-3.73 | 0.45 | -1.15 | 0.03 | 1.59 |
| 5-Acetylamino-6-amino-3-methyluracil | 199.0828-1.34 | 0.45 | -1.15 | 0.01 | 2.00 |
| Decarbamoylneosaxitoxin | 290.1583-4.50 | 0.46 | -1.11 | 0.03 | 1.55 |
| Polypropylene glycol | 152.1283-3.48 | 0.47 | -1.09 | 0.02 | 1.64 |
| 6-hydroxyindolelactic acid | 222.0761-6.52 | 0.47 | -1.09 | 0.04 | 1.36 |
| N/A | 152.1283-4.29 | 0.48 | -1.06 | 0.04 | 1.45 |
| Polypropylene glycol | 135.1017-4.28 | 0.48 | -1.05 | 0.04 | 1.41 |
| 2-Ketohexanoic acid | 131.0703.4.59 | 0.49 | -1.03 | 0.03 | 1.55 |
| N/A | 157.0837-3.47 | 0.49 | -1.02 | 0.03 | 1.55 |
| N/A | 337.7002-6.70 | 2.04 | 1.03 | 0.03 | 1.48 |
| N/A | 137.9650-4.83 | 2.05 | 1.03 | 0.04 | 1.40 |
| N/A | 361.1520-8.03 | 2.17 | 1.12 | 0.03 | 1.52 |
| N/A | 192.9760.1.61 | 2.18 | 1.12 | 0.01 | 2.15 |
| N/A | 126.9718-4.91 | 2.20 | 1.14 | 0.05 | 1.33 |
| N/A | 153.9476-1.62 | 2.42 | 1.27 | 0.01 | 2.20 |
| Vanillin | 153.0543-8.25 | 2.62 | 1.39 | 0.01 | 2.29 |
| N/A | 100.0755-1.43 | 2.65 | 1.40 | 0.02 | 1.68 |
| [4-formyl-8,9-dihydroxy-8a-(hydroxymethyl)-4,6a,6b,11,11,14b-hexamethyl-1,2,3,4,4a,5,6,6a,6b,7,8,8a,9,10,11,12,12a,14,14a,14b-icosahydropicen-3-yl]oxidanesulfonic acid | 569.3165-6.53 | 2.79 | 1.48 | 0.05 | 1.32 |
| N/A | 139.9236-1.41 | 2.83 | 1.50 | 0.01 | 2.05 |
| Bupropion | 240.1143-1.52 | 2.96 | 1.56 | 0.00 | 2.38 |
| Citrulline | 158.0927-1.43 | 3.08 | 1.62 | 0.01 | 2.22 |
| (2-{[3-(2,4-dihydroxy-3-methoxyphenyl)prop-2-enoyl]oxy}ethyl)trimethylazanium | 296.1506-6.55 | 3.19 | 1.67 | 0.01 | 1.89 |
| Tranexamic Acid | 158.1173-1.24 | 3.23 | 1.69 | 0.03 | 1.46 |
| N/A | 126.9719-4.06 | 3.43 | 1.78 | 0.03 | 1.46 |
| Naratriptan | 318.1654-6.62 | 3.45 | 1.79 | 0.00 | 2.35 |
| N/A | 168.0631-1.15 | 3.69 | 1.88 | 0.04 | 1.40 |
| Arginylasparagine | 289.1615-6.13 | 3.77 | 1.91 | 0.04 | 1.38 |
| N/A | 520.3349-6.42 | 4.17 | 2.06 | 0.03 | 1.51 |
| {15-[(3E)-5,6-dimethylhept-3-en-2-yl]-10-hydroxy-2,16-dimethyl-8,19-dioxahexacyclo[9.7.1.0]nonadecan-5-yl}oxidanesulfonic acid | 525.2893-6.42 | 4.32 | 2.11 | 0.02 | 1.68 |
| 4-{5-[(4,5-dihydroxy-6-methyloxan-2-yl)oxy]-11,17-dihydroxy-2,15-dimethyltetracyclo[8.7.0.0]heptadecan-14-yl}-2,5-dihydrofuran-2-one | 503.3029-6.42 | 4.63 | 2.21 | 0.02 | 1.62 |
| N/A | 209.1267-4.98 | 4.74 | 2.24 | 0.00 | 3.50 |
| N/A | 477.3128-6.30 | 5.79 | 2.53 | 0.02 | 1.63 |
| Netilmicin | 476.3060-6.30 | 5.83 | 2.54 | 0.04 | 1.45 |
| N/A | 433.2849-6.17 | 5.85 | 2.55 | 0.03 | 1.52 |
| Citronellyl beta-sophoroside | 481.2642-6.31 | 6.10 | 2.61 | 0.02 | 1.71 |
| 2-Dodecylbenzenesulfonic acid | 344.2268.5.83 | 6.13 | 2.62 | 0.04 | 1.37 |
| 4-[(2S,15S)-2,15-dimethyl-16-(sulfooxy)tetracyclo[8.7.0.0]heptadecan-14-yl]pent-4-enoic acid | 437.2382-6.17 | 6.92 | 2.79 | 0.02 | 1.66 |
| N/A | 388.2564-6.02 | 7.47 | 2.90 | 0.04 | 1.35 |
| N/A | 432.2778.6.17 | 7.76 | 2.96 | 0.04 | 1.43 |
| Physoperuvine | 159.1488-1.44 | 7.96 | 2.99 | 0.02 | 1.79 |
| N/A | 349.1812-5.83 | 8.32 | 3.06 | 0.02 | 1.62 |
| Paramethasone | 393.2088-6.02 | 9.31 | 3.22 | 0.01 | 1.87 |
| Octaethylene glycol | 371.2269.6.02 | 11.79 | 3.56 | 0.02 | 1.74 |
| N/A | 372.2604-5.83 | 14.78 | 3.89 | 0.01 | 1.89 |
| N/A | 319.1370-5.70 | 18.24 | 4.19 | 0.04 | 1.45 |
| N/A | 416.2825-6.02 | 18.72 | 4.23 | 0.04 | 1.38 |
| N/A | 314.1824-5.71 | 20.64 | 4.37 | 0.04 | 1.45 |
| N/A | 342.2127-5.70 | 38.20 | 5.26 | 0.02 | 1.70 |

|  |  |  |  |  |  |
| --- | --- | --- | --- | --- | --- |
| **Timepoint 5** | | | | | |
| **ID** | **Mass** | **FC** | **log2(FC)** | **raw.pval** | **-log10(p)** |
| N1,N10-Dicoumaroylspermidine* | 438.2373-6.31 | 0.15 | -2.75 | 0.02 | 1.69 |
| N/A | 569.3165-6.53 | 0.19 | -2.36 | 0.03 | 1.52 |
| Esmolol* | 296.1844-6.62 | 0.23 | -2.11 | 0.03 | 1.52 |
| N/A | 318.1654-6.62 | 0.25 | -1.99 | 0.02 | 1.63 |
| 6-({1,7-dihydroxy-2,15-dimethyl-5-oxo-14-[(3E)-4,5,6-trimethylhept-3-en-2-yl]tetracyclo[8.7.0.0]heptadec-9-en-8-yl}oxy)-3,4,5-trihydroxyoxane-2-carboxylic acid* | 652.4050-6.70 | 0.25 | -1.98 | 0.04 | 1.42 |
| N/A | 326.7096-6.70 | 0.35 | -1.52 | 0.01 | 1.85 |
| Prostaglandin H2* | 335.2238-6.69 | 0.41 | -1.29 | 0.04 | 1.39 |
| N/A | 357.7386-6.78 | 0.42 | -1.24 | 0.03 | 1.49 |
| N/A | 290.1583-4.50 | 0.43 | -1.21 | 0.01 | 1.87 |
| Ferulic acid* | 195.0653-7.37 | 0.45 | -1.17 | 0.04 | 1.39 |
| N/A | 348.7227-6.78 | 0.47 | -1.10 | 0.00 | 2.35 |
| N/A | 340.2096-6.78 | 0.47 | -1.09 | 0.01 | 1.82 |
| p-Aminobenzoic acid* | 155.0815-1.22 | 0.48 | -1.05 | 0.00 | 2.45 |
| N/A | 233.1260-2.12 | 2.17 | 1.12 | 0.02 | 1.80 |
| N/A | 158.0927-1.43 | 2.21 | 1.14 | 0.02 | 1.61 |
| 2-Phenylethyl 3-methylbutanoate* | 207.1386-6.89 | 2.65 | 1.41 | 0.02 | 1.74 |
| 3-Dehydrocarnitine* | 160.0968-1.18 | 3.09 | 1.63 | 0.02 | 1.82 |
