# Supplementary material for: Identification of food and nutrient components as predictors of Lactobacillus colonization
Source: Front Nutr. 2023 Apr 21;10:1118679. doi: 10.3389/fnut.2023.1118679 (PMC10160632; doi:10.3389/fnut.2023.1118679)
Supplement: Supplementary file 5 [file Table_5.DOCX]

Table S5. Growth kinetics, µmax and doubling time (DT) of select heterofermentative, homofermentive, and facultative heterofermentative *Lactobacillus* species when grown in MRS with Tween 80 and vehicle control DMSO (MRS-T-DMSO), MRS without Tween 80 (MRS-NT), MRS-NT supplemented with 0.1% oleic acid (MRS-O), MRS-NT supplemented with 0.1% erucic acid (MRS-E), MRS with Tween 80 (MRS-T). ^a,b,c,d,e^p>0.05, NG = No Growth detected

|  | **µmax** | | | | | **DT** | | | | |
| --- | --- | --- | --- | --- | --- | --- | --- | --- | --- | --- |
|  | **MRS-NT** | **MRS-D** | **MRS-T** | **MRS-O** | **MRS-E** | **MRS-NT** | **MRS-D** | **MRS-T** | **MRS-O** | **MRS-E** |
| *L. johnsonii* N6.2 | NG | 0.19 ± 0.02^a^ | 0.24 ± 0.02^b^ | 0.17 ± 0.03^a^ | 0.09 ± 0.02^c^ | NG | 1±0.08^a^ | 1.34±0.05^b^ | 0.85±0.06^a^ | 1.52±0.16^b^ |
| *L. johnsonii* ATCC 33200 | NG | 0.1 ± 0.01^a^ | 0.24 ± 0.01^b^ | 0.27 ± 0.01^c^ | 0.08 ± 0.01^d^ | NG | 1.05±0.03^a^ | 1.33±0.05^b^ | 0.77±0.01^c^ | 0.29±0.01^d^ |
| *L. amylovorus* ATCC 33620 | NG | 0.17 ± 0.01^a^ | 0.26 ± 0.03^b^ | NG | 0.17 ± 0.02^a^ | NG | 0.86±0.01^d^ | 1.34±0.06^a^ | NG | 1.17±0.12^a^ |
| *Lig. murinus* 35020 | NG | 0.13 ± 0.01^a^ | 0.18 ± 0.01^b^ | 0.21 ± 0.02^b^ | 0.14 ± 0.01^a^ | NG | 0.71±0.03^a^ | 1.01±0.03^b^ | 0.71±0.03^a^ | 0.96±0.05^b^ |
| *Lim. fermentum* 14391 | 0.42 ± 0.01^a^ | 0.24 ± 0.02^b^ | 0.14 ± 0.01^cd^ | 0.16 ± 0.01^c^ | 0.15 ± 0.01^c^ | 0.91±0.02^a^ | 1.07±0.03^b^ | 0.83±0.02^c^ | 1.64±0.04^d^ | 1.38±0.02^e^ |
| *Lim. reuteri* TD1 | NG | 0.12 ± 0.01^a^ | 0.31 ± 0.02^b^ | 0.20 ± 0.01^c^ | 0.19 ± 0.01^c^ | NG | 0.92±0.04^a^ | 0.68±0.03^b^ | 0.46±0.04^c^ | 0.86±0.11^a^ |
| *Lac. plantarum* 25302 | 0.12 ± 0.01^a^ | 0.17 ± 0.01^b^ | 0.21 ± 0.02^c^ | 0.21 ± 0.01^c^ | 0.16 ± 0.02^b^ | 1.16±0.08^a^ | 1.09±0.11^a^ | 1.36±0.04^b^ | 0.78±0.01^c^ | 1.04±0.04^a^ |
| *Lcc. casei* ATCC 334 | 0.11 ± 0.01^a^ | 0.14 ± 0.01^bc^ | 0.15 ± 0.01^b^ | 0.13 ± 0.01^c^ | 0.34 ± 0.01^d^ | 1.04±0.05^ad^ | 1.12±0.03^ab^ | 1.13±0.02^b^ | 0.91±0^c^ | 1.01±0.03^d^ |
