# Supplementary material for: Identification of food and nutrient components as predictors of Lactobacillus colonization
Source: Front Nutr. 2023 Apr 21;10:1118679. doi: 10.3389/fnut.2023.1118679 (PMC10160632; doi:10.3389/fnut.2023.1118679)

Figure S1. Data analysis design and stratification strategy.


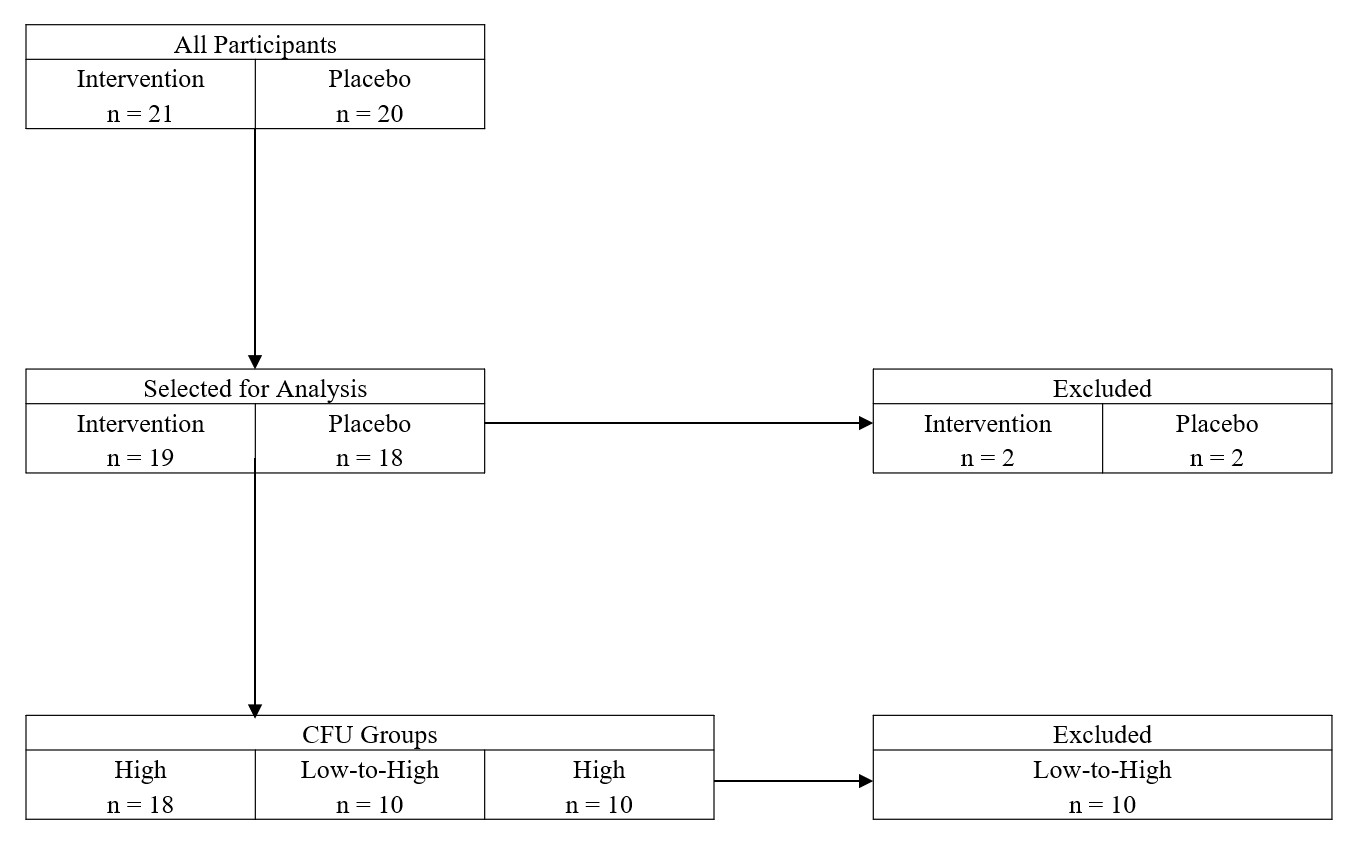


Figure S2. Principal component regression considering the Log(CFU/g wet stool) LAB and a) vegetables dimension 2 (R^2^ = 0.52, p = 1.35e-05) and b) meats dimension 1 (R^2^ = 0.59, p = 1.73e-06).

**A** **B**


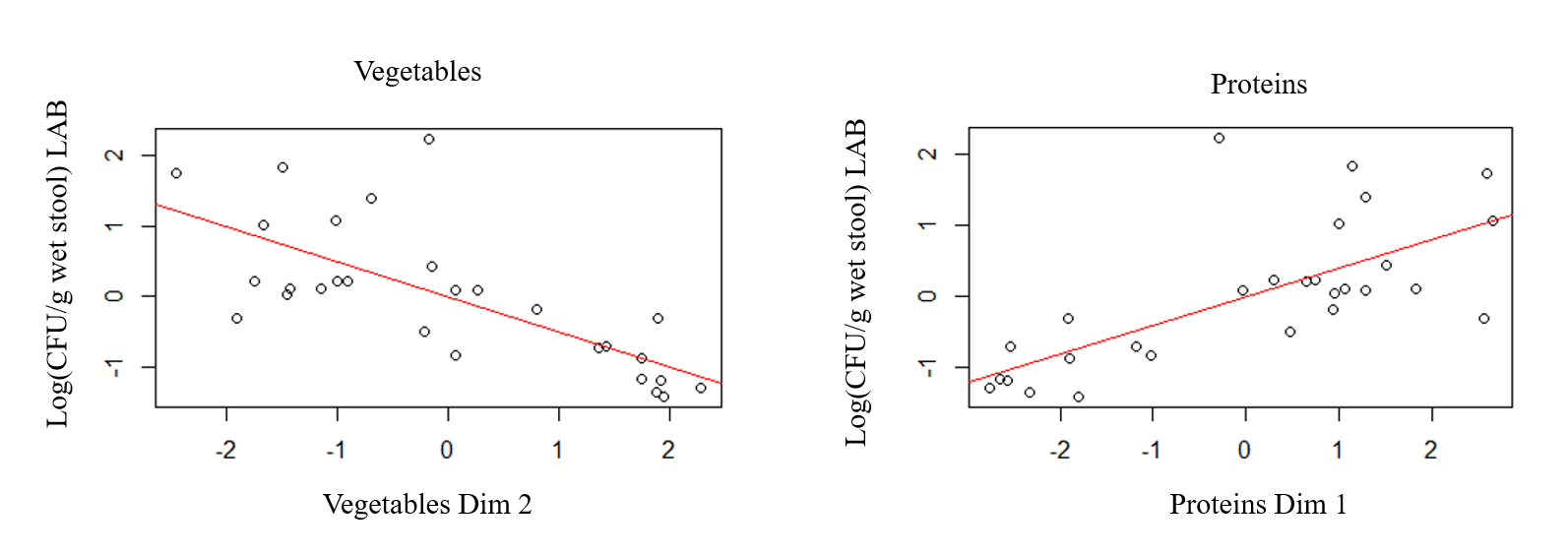


Proteins (Dim 1))

Vegetables (Dim 2))

Figure S3. PCA of serum metabolites were performed using Metaboanalyst® and represented as PCA plots (left) or volcano plots (right). Metabolites were analyzed by A) Intervention vs Placebo (“Ljo” and “Placebo”), B) H-LAB vs L-LAB (“H” and “L”), C) H-LAB and L-LAB at Timepoint 1, D) H-LAB and L-LAB at Timepoint 2, E) H-LAB and L-LAB at Timepoint 3, F) H-LAB and L-LAB at Timepoint 4, G) H-LAB and L-LAB at Timepoint 5.

**A**


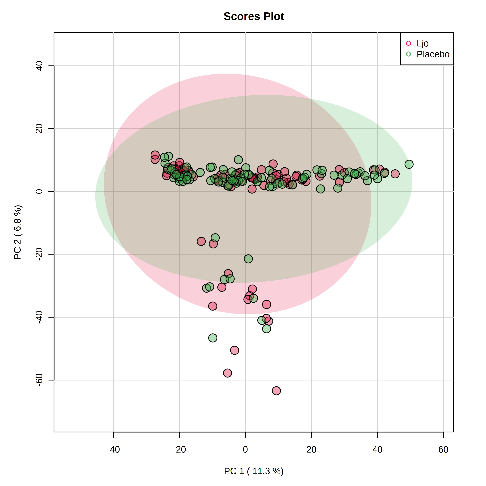

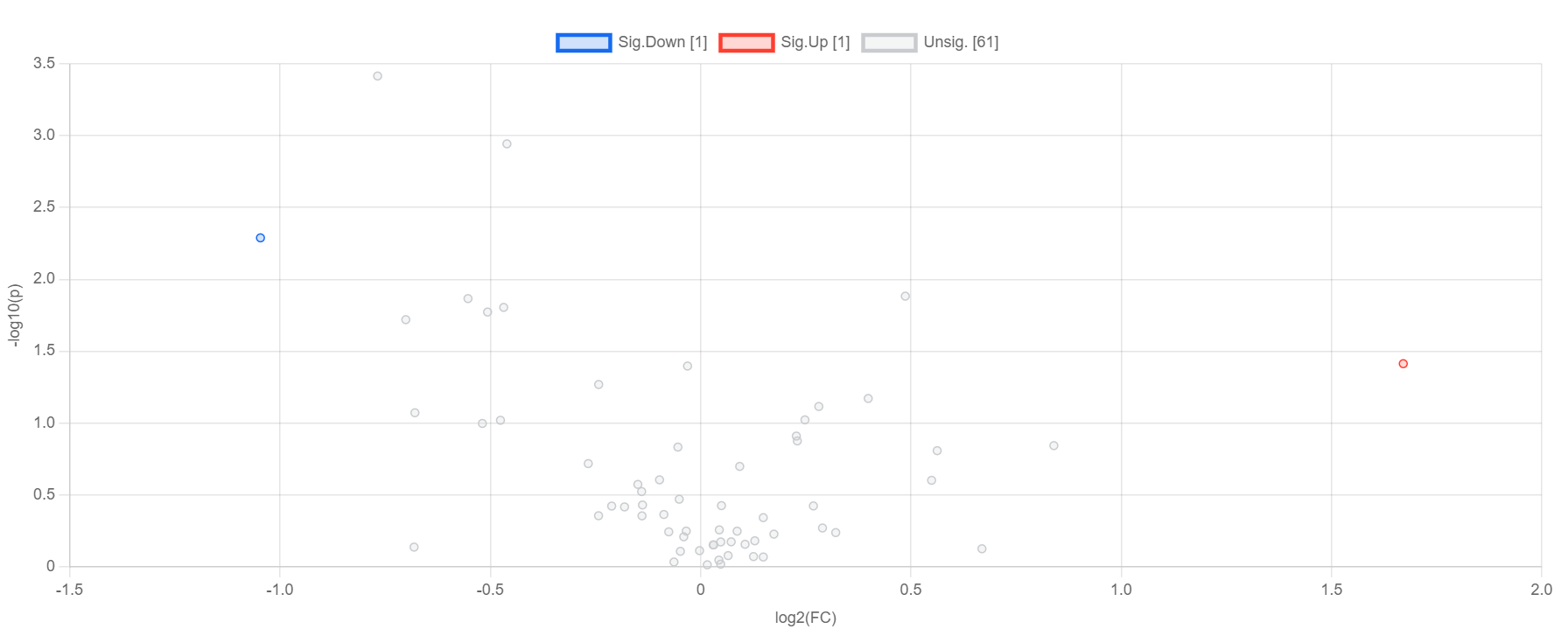


**B**


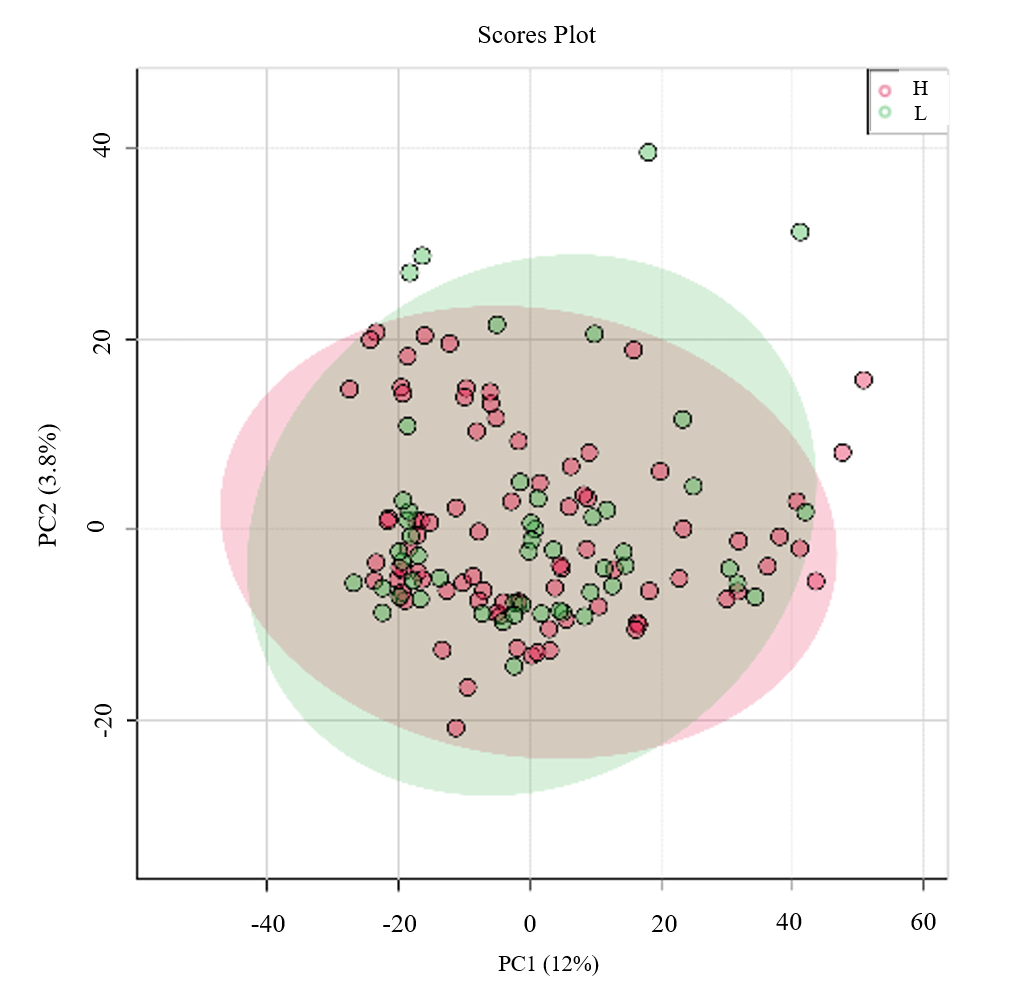

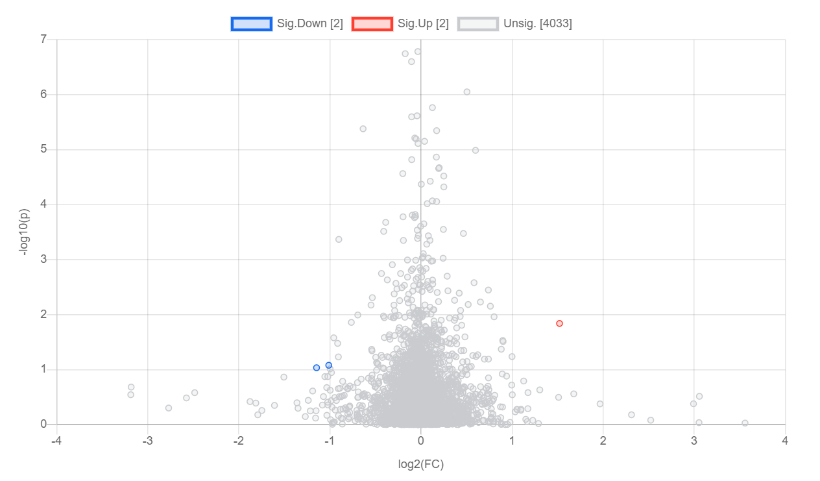


**C**


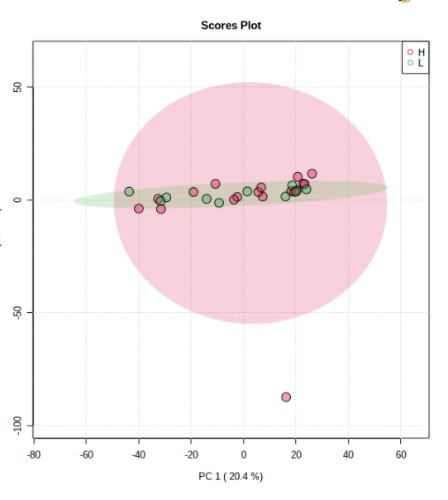

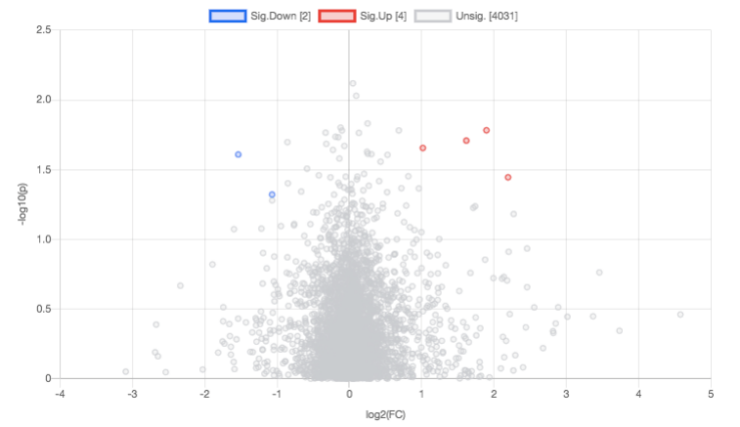


**D**


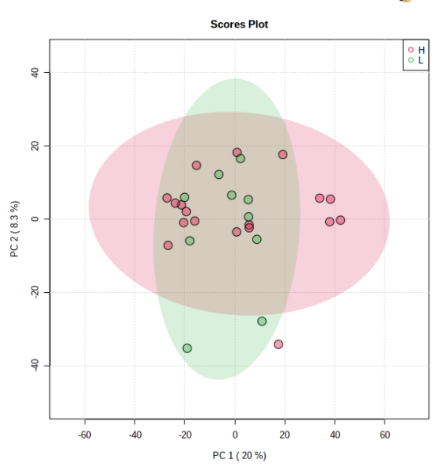

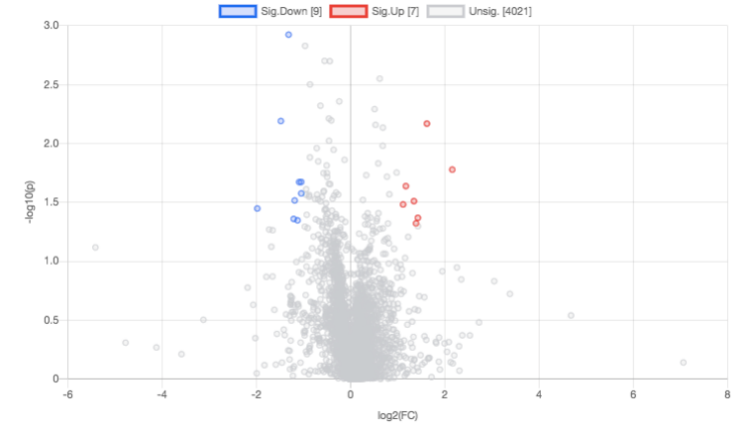


**E**


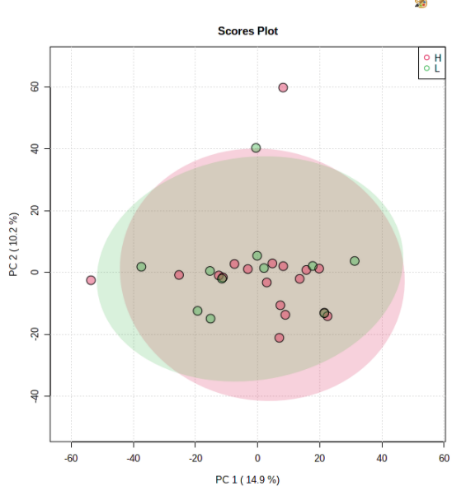

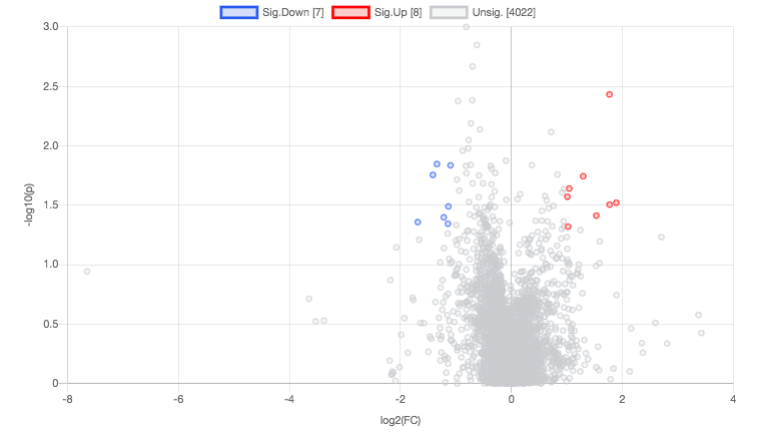


**F**


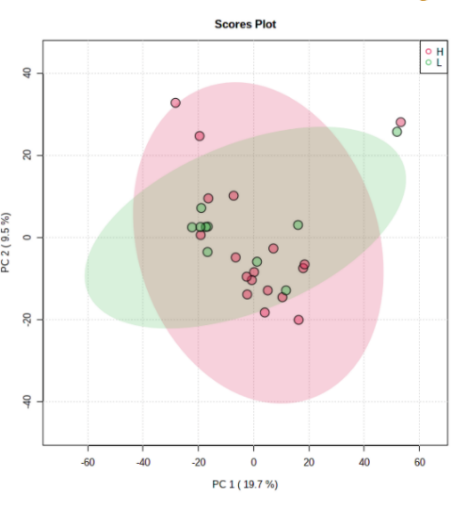

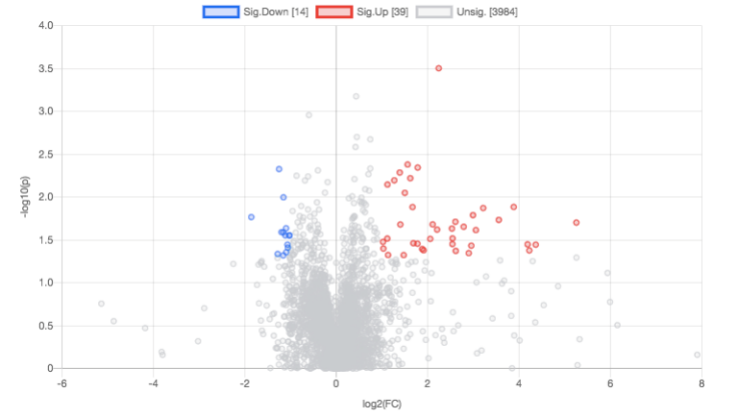


**G**


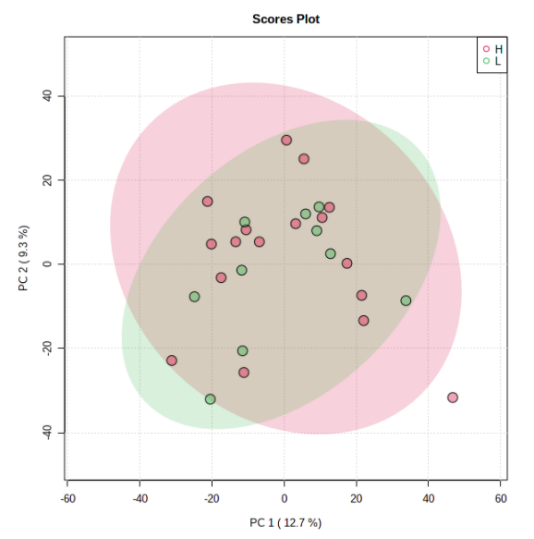

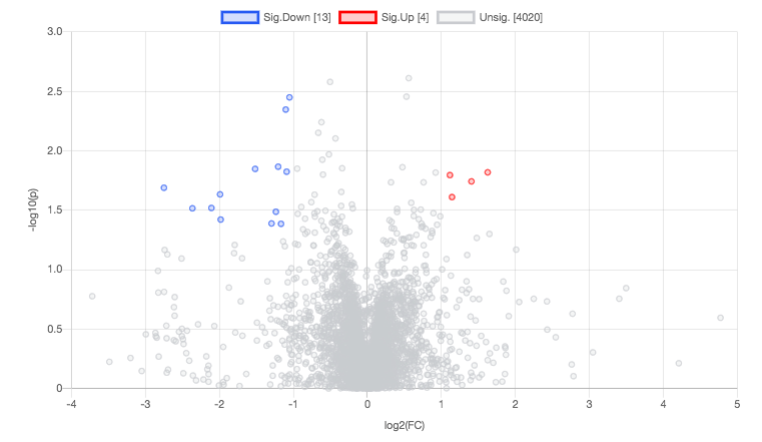

Supplement: Supplementary file 6 [file Data_Sheet_1.DOCX]
